# Lincosamide Synthetase—A Unique Condensation System Combining Elements of Nonribosomal Peptide Synthetase and Mycothiol Metabolism

**DOI:** 10.1371/journal.pone.0118850

**Published:** 2015-03-05

**Authors:** Jiri Janata, Stanislav Kadlcik, Marketa Koberska, Dana Ulanova, Zdenek Kamenik, Petr Novak, Jan Kopecky, Jitka Novotna, Bojana Radojevic, Kamila Plhackova, Radek Gazak, Lucie Najmanova

**Affiliations:** 1 Institute of Microbiology, Academy of Sciences of the Czech Republic, v.v.i., Prague, Czech Republic; 2 Oceanography Section, Science Research Center, Kochi University, IMT-MEXT, Kohasu, Oko-cho, Nankoku, Kochi, 783–8505, Japan; University Paris South, FRANCE

## Abstract

In the biosynthesis of lincosamide antibiotics lincomycin and celesticetin, the amino acid and amino sugar units are linked by an amide bond. The respective condensing enzyme lincosamide synthetase (LS) is expected to be an unusual system combining nonribosomal peptide synthetase (NRPS) components with so far unknown amino sugar related activities. The biosynthetic gene cluster of celesticetin was sequenced and compared to the lincomycin one revealing putative LS coding ORFs shared in both clusters. Based on a bioassay and production profiles of *S. lincolnensis* strains with individually deleted putative LS coding genes, the proteins LmbC, D, E, F and V were assigned to LS function. Moreover, the newly recognized N-terminal domain of LmbN (LmbN-CP) was also assigned to LS as a NRPS carrier protein (CP). Surprisingly, the homologous CP coding sequence in celesticetin cluster is part of *ccbZ* gene adjacent to *ccbN*, the counterpart of *lmbN*, suggesting the gene rearrangement, evident also from still active internal translation start in *lmbN*, and indicating the direction of lincosamide biosynthesis evolution. The *in vitro* test with LmbN-CP, LmbC and the newly identified *S. lincolnensis* phosphopantetheinyl transferase Slp, confirmed the cooperation of the previously characterized NRPS A-domain LmbC with a *holo*-LmbN-CP in activation of a 4-propyl-L-proline precursor of lincomycin. This result completed the functional characterization of LS subunits resembling NRPS initiation module. Two of the four remaining putative LS subunits, LmbE/CcbE and LmbV/CcbV, exhibit low but significant homology to enzymes from the metabolism of mycothiol, the NRPS-independent system processing the amino sugar and amino acid units. The functions of particular LS subunits as well as cooperation of both NRPS-based and NRPS-independent LS blocks are discussed. The described condensing enzyme represents a unique hybrid system with overall composition quite dissimilar to any other known enzyme system.

## Introduction

**Fig 1:**
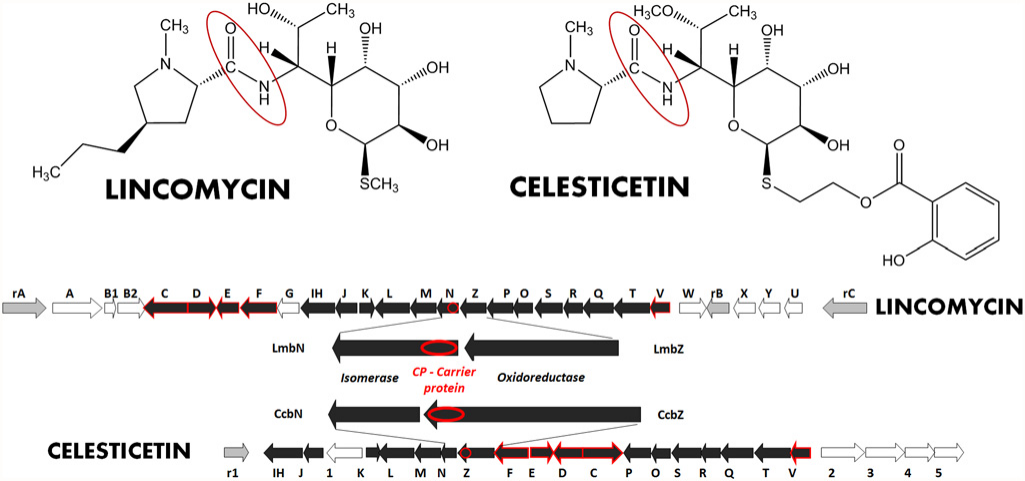
Structures and biosynthetic gene clusters of natural lincosamides. The resistance genes are marked in grey, the genes homologous in both gene clusters are black. The genes highlighted by red line exhibit inactivation pattern enabling their assignment to condensation reaction, i.e. formation of amide bond (in red oval in the structures).

Lincomycin and celesticetin are two structurally related natural compounds (Fig. 1), representatives of the small group of lincosamide antibiotics. Lincosamides are composed of amino sugar and amino acid moieties and their biosynthetic pathways thus combine some features of aminoglycoside and peptide antibiotics biosyntheses [1,2]. The condensation of the amino sugar and amino acid precursors catalyzed by lincosamide synthetase (LS; this and other acronyms listed in Table 1) is the crucial, but so far the less clarified part of the lincosamide biosynthesis. A previous study of the lincomycin biosynthetic pathway [3] suggested that the appropriate condensation enzyme N-demethyllincomycin synthetase (NDLS) is a multimeric complex, though the individual components were not identified. However, according to the nature of the condensed precursors, a hybrid system combining nonribosomal peptide synthetase (NRPS) elements with so far unknown amino sugar related activities can be expected.

**Table 1:** Abbreviations of specific compounds and proteins.

NRPS	nonribosomal peptide synthetase
LS	lincosamide synthetase
CP	carrier protein
A-domain	adenylation domain
PPL	4-propyl-L-proline
NDLS	N-demethyllincomycin synthetase
MTL	methylthiolincosamide
ID	isomerase domain
PPTase	phosphopantetheinyl transferase
C8	octose
MDMPI	mycothiol maleylpyruvate isomerase

Indeed, the only identified and characterized component of NDLS is LmbC protein, a stand-alone adenylation domain (A-domain) highly specific for activation of 4-propyl-L-proline (PPL), an unusual amino acid precursor of lincomycin [4]. Homologous CcbC protein specifically activates proteinogenic L-proline (but not PPL) in the biosynthesis of celesticetin. A-domain is an obligatory component of NRPS initiation module in the biosynthesis of peptide compounds, together with a carrier protein (CP). The CP function is tightly connected with the activation step as the activated amino acid is transferred from A-domain to CP. The participation of a CP in LS system is thus reasonably expected. Reflecting the functional connection, the CP domain is frequently fused with the respective A-domain [5]. However, this does not hold for LmbC/CcbC. The CP can alternatively exist as a stand-alone element. The sequence of lincomycin biosynthetic gene cluster is available (EU124663; [6]) but no ORF coding for an autonomous CP has been identified. The CP coding sequence thus should be searched as an integral part of other biosynthetic genes.

The prediction of remaining LS components is rather more complicated. Since the amino acid precursor in lincosamide biosynthesis is condensed to amino sugar, instead of another amino acid unit as in non-ribosomally synthesized peptides, the LS overall subunit composition should reflect this specificity and differ from that of NRPS. The side chains of core amino acid residues in NRPS-synthesized peptides are often glycosylated [7], but the attachment of amino sugar to amino acid α-carboxyl group by amide bond is relatively rare in secondary metabolites. Besides lincosamides, it is known e.g. from the biosynthesis of streptothricin [8] or metabolism of mycothiol and its conjugates forming the detoxification system specific for actinobacteria [9] but widespread within this group.

To search for missing putative LS components, the comparative analysis of lincomycin and celesticetin biosynthetic gene clusters can provide fundamental initial information. The appropriate gene clusters should reflect both, the high similarity of compared lincosamide biosynthetic pathways as well as compound specific features. Lincomycin incorporates an unusual amino acid precursor PPL (instead of L-proline in celesticetin) synthesized by a specific pathway from L-tyrosine [10]. On the other hand, the celesticetin structure contains an additional specific salicylate building unit connected to the amino sugar moiety. Genes encoding the biosynthesis of these specific precursors or compound specific modifications do not have their counterparts in the other compared gene cluster, while the remaining biosynthetic genes are present as homologous pairs in both clusters. These shared genes thus encode first the biosynthesis of amino sugar precursor core, second the only common methylation step and, last but not least, the searched subunits of LS.

The LmbJ/CcbJ homologous pair of proteins was recently proved to catalyze the above mentioned common methylation step [11,12]. The functions of several enzymes involved in the biosynthesis of methylthiolincosamide (MTL), the amino sugar precursor of lincomycin, have already been elucidated: Proteins encoded by genes *lmbO*, *lmbN*, *lmbR* and *lmbK* were recently demonstrated to catalyze four out of the five initial MTL biosynthetic steps [13,14]. Moreover, the sequence analysis makes it possible to fairly predict also the functions of other MTL biosynthetic genes. These genes show significant homology to genes from the biosyntheses of aminoglycoside antibiotics and amino sugars in general [1,2].

In this paper, six proteins or protein parts were identified as putative LS components based on comparative analysis of lincomycin and celesticetin biosynthetic gene clusters, inactivations of corresponding lincomycin biosynthetic genes and subsequent bioassays of mutant strains combined with LC-MS analyses of fermentation media. Together with previously published results [4], we completed the functional evidence of the two NDLS components resembling features of the NRPS system, i.e. A-domain and CP domain, and proposed the functions of remaining putative LS subunits, forming together the unique hybrid condensation system.

## Materials and Methods

### Bacterial strains

The type strains *Streptomyces lincolnensis* ATCC 25466 and *Streptomyces caelestis* ATCC 15084 producing lincomycin and celesticetin, respectively, were used. The latter strain is a source of celesticetin biosynthetic gene cluster sequence (S1 Document). All *S. lincolnensis* mutant strains were derived from *S. lincolnensis* ATCC 25466. *S. coelicolor M145* was used as a host strain for heterologous expression of *lmbN* forms for immunodetection assays. *Kocuria rhizophila* ATCC 9341 was used as lincomycin-sensitive strain for the bioassay. Routine DNA manipulations were performed in *E. coli* XL1 Blue MR^b^ (Stratagene). The heterologous expression of *S. lincolnensis* genes was performed in *E. coli* BL21(DE3) (Novagen). *E. coli* ET12567, BW25113 and DH5 strains were used for gene inactivation.

### Gene inactivation

Selected lincomycin biosynthetic genes were inactivated by introduction of apramycin resistance cassette using the REDIRECT technology kit for PCR targeting [15]. The kit was obtained from Plant Bioscience Limited (Norwich Research Park, Colney, Norwich). Inactivation and checking primers are listed in S1 Table. For construction of Δ*lmbN*, *lmbN_*Δ*CP*, *lmbN_*Δ*ID*, Δ*lmbIH*, Δ*lmbQ*, Δ*lmbT* and Δ*lmbV* mutants, the inactivation cassette was replaced by 81nt in frame scar to avoid the polar effect on transcription of a downstream gene.

### Cultivation of streptomycete strains

For the bioassay, *S. lincolnensis* strains were cultivated on GYM *Streptomyces* agar [16] at 30°C for 5 and 10 days. For chemical complementation the agar was supplemented with MTL and/or PPL (200 μgmL^-1^). The cultivation was carried out on 96-well Terasaki plates to give uniform 200 μL agar plugs.

For LC-MS analysis of fermentation media, the seed culture of *S. lincolnensis* strains was prepared by inoculating spores into 50 ml of YEME medium [17] in 250 mL Erlenmeyer flasks and incubation at 30°C for 24 h. Then, 1.25 mL of the seed culture was inoculated into 25 mL of AVM medium [18] in 250 mL Erlenmeyer flasks and incubated at 30°C for 60 or 120 h. The cells were centrifuged at 4000 g at 4°C for 10 min and the supernatant was used immediately or stored at -20°C.

For immunodetection assays the seed culture of *Streptomyces* strains was prepared by inoculation from GYM plates into 50 mL of the YEME medium without sucrose and incubated in 500 mL flat-bottom boiling flasks at 28°C. A 2 mL volume of 30 h seed culture of *S. lincolnensis* was inoculated into 40 mL of AVM medium and incubated in 500 mL flat-bottom boiling flasks at 28°C for 48, 72, 96, 120 and 144 h. The 48 h seed culture was directly used for *S. coelicolor*. The cells were harvested by centrifugation, washed twice with 40 mL of TS-8 buffer (20 mM Tris-HCl, 100 mM NaCl, pH 8.0) and stored at -20°C

### Bioassay and chemical complementation

The agar plugs with 5- or 10-day mycelium were transferred at least in triplicates on plates overlaid with sensitive strain *Kocuria rhizophila*, the plates were cultivated at 30°C overnight, and the antibiotic production was detected by growth inhibition zones around the plugs.

### LC-MS analysis of lincomycin and its precursors in fermentation media

*Standards*: Lincomycin was purchased from Sigma-Aldrich (Germany) and the standards of MTL, PPL, 4-butyl-L-proline and N-demethyllincomycin were prepared as described previously [19,20]. The standard solutions were prepared in water at the concentration of 1 mgmL^-1^ and spiked in the matrix or sample at the required concentration.

*Sample and matrix preparation*: For analyses of lincomycin and MTL, the samples (supernatants of cultivation broths) were cleaned-up by solid phase extraction with Oasis cartridges (Waters); for details, see S2 Table part A. The extract was reconstituted in 50% methanol, spiked with internal standard (only when quantification was performed) and analyzed. For analyses of PPL, the extraction step was omitted and the analyte was determined in the cultivation broth spiked with an internal standard. The matrix for calibration and dilution of too concentrated samples was prepared by extraction of analyte-free spent cultivation broth.

*Conditions of LC-MS analyses*: LC-MS analyses were performed on Acquity UPLC system with LCT premier XE time-of-flight mass spectrometer (Waters, USA). Five μL of sample was loaded onto the LC column Acquity UPLC BEH C18 (50 mm × 2.1 mm I.D., particle size 1.7μm, Waters) kept at 30°C and eluted with a two-component mobile phase under a linear gradient at the flow rate of 0.4 mLmin^-1^. The mass spectrometer operated in the “W” mode with capillary voltage set at +2800 V, cone voltage +40 V, desolvation gas temperature, 350°C; ion source block temperature, 120°C; cone gas flow, 50 Lh^-1^; desolvation gas flow, 800 Lh^-1^; scan time of 0.1 s; inter-scan delay of 0.01 s. The data were processed by MassLynx V4.1 (Waters). For details, see S2 Table parts B and C.

### Heterologous production of LmbN protein forms in *E. coli*

Each gene was PCR amplified from LK6 cosmid [6] using the primer pairs according to S3 Table and inserted into an appropriate vector. The resulting construct was used to produce soluble protein in *E. coli* BL21(DE3). The proteins were produced and purified as was described for LmbC [4] with specific details summarized in S3 Table. Buffer exchange and protein storage conditions are described later where it is appropriate.

### Preparation of rabbit polyclonal anti-LmbN antibody

Purified LmbN prepared using construct 1 (S3 Table) was dialyzed overnight against TS-8 buffer and delivered to BioGenes company (Germany). Prepared rabbit polyclonal anti-LmbN antibody in the form of serum was used for immunodetection experiments.

### Preparation of *S. coelicolor* M145 mutant strains

The *lmbN* gene was PCR amplified from the LK6 cosmid using the two primer pairs (S1 Table): ExN_CHisF/ExN_coelR for full length *lmbN* and ExNStopF/ExN_coelR for mutant *lmbN* with stop codon introduced immediately downstream of the natural start codon. The amplified sequences were inserted via the *Nde*I and *Xho*I restriction sites into an integrative vector pIJ10257 [21]. Resulting plasmids were introduced into non-methylating strain *E. coli* ET12567/pUZ8002 and then introduced into *S. coelicolor* M145 genome via intergeneric conjugal transfer. Exconjugants were selected with hygromycin (0.1 gL^-1^).

### Immunodetection assay of LmbN protein forms

The cells of *S. lincolnensis* or *S. coelicolor* (200 μL of wet cell mass) were stirred in 1 mL of TS-8 buffer with complete mini EDTA-free protease inhibitor cocktail (Roche) and disrupted by sonication. The cell lysates were separated by 10% SDS-PAGE and Western blotted onto a nitrocellulose membrane (Bio-Rad). The membranes were incubated for 1 h at 24°C with a blocking solution (1% (w/v) non-fat dried milk, 100 mM maleic acid, 150 mM NaCl, pH 7.5) on an orbital shaker, then washed 5x 15 min at 24°C in washing buffer (PBS containing 0.05% (v/v) Tween-20, pH 7.4) and incubated for 1.5 h with washing buffer containing rabbit polyclonal anti-LmbN antibody and 1% (w/v) non-fat dried milk. The membranes were then washed as previously, incubated for 1.5 h with washing buffer containing 1% (w/v) non-fat dried milk and horseradish peroxidase-conjugated monoclonal anti-rabbit IgG (Sigma-Aldrich) and finally washed again. Antibody complexes were detected using Immobilon Western HRP substrate (Millipore) and visualized on Hyperfilm ECL (Amersham).

### Phosphopantetheinylation of apo-LmbN-CP in vivo

LmbN-CP was prepared by co-production with Slp using construct 3 (S3 Table) and construct pslp1 (S2 Document). The co-production was verified by SDS-PAGE. Purified LmbN-CP was dialyzed overnight against 50 mM Tris-HCl (pH 8.8) and used for the aminoacylation assay. Moreover, the dialyzed LmbN-CP diluted with 50 mM Tris-HCl (pH 8.8) to a concentration of 1mgmL^-1^ (100 μL) was kept at -80°C prior to the LC-MS analysis.

### Phosphopantetheinylation of apo-LmbN-CP in vitro

LmbN-CP was produced using the construct 3 (S3 Table). PPTase Slp was prepared using the construct pslp2 as described in S2 Document. Each protein, i.e. LmbN-CP and Slp, was then dialyzed overnight against 50 mM Tris-HCl, pH 8.8. *In vitro* phosphopantetheinylation assay contained 75 mM Tris-HCl, 150 μM LmbN-CP, 2 μM Slp, 8 mM MgCl_2_, 2.5 mM CoA, 2 mM TCEP, pH 8.8, in a final volume of 200 μL. The reaction was initiated by addition of Slp and the reaction mixture was incubated at 28°C for 50 minutes. 100 μL of the reaction mixture was then kept at -80°C prior to LC-MS analysis. 67 μL of the reaction mixture was immediately used for aminoacylation of *holo*-LmbN-CP by LmbC.

### Aminoacylation of holo-LmbN-CP by LmbC

LmbC was produced as described previously [4] with additional 20% (v/v) glycerol in all buffers. The reaction mixture with *in vivo* phosphopantetheinylated LmbN-CP contained 100 μM LmbN-CP, 1.2 mM ATP, 2 mM MgCl_2_, 10% (v/v) glycerol, 2 mM TCEP, 3 mM PPL, 0.6 μM LmbC, 75 mM Tris-HCl, pH 8.8, in a final volume of 100 μL. The reaction mixture with *in vitro* phosphopantetheinylated LmbN-CP contained 67 μL of the *in vitro* phosphopantetheinylation reaction mixture, 1.2 mM ATP, 5.4 mM MgCl_2_, 10% (v/v) glycerol, 2 mM TCEP, 3 mM PPL, 0.6 μM LmbC, 75 mM Tris-HCl, pH 8.8 in a final volume of 100 μL. The assays were initiated by addition of LmbC, incubated at 28°C for 50 minutes and then kept at -80°C prior the LC-MS analysis of LmbN-CP.

### LC-MS analysis of LmbN-CP samples

The LmbN-CP protein samples were diluted using 0.1% aqueous solution of trifluoroacetic acid to a concentration of 0.1 mgmL^-1^ and one μl of each sample was loaded onto a reverse phased trap Acclaim PepMap 100 C18 column (0.1 × 20 mm, 5 μm, 100 A, Thermo Fisher Scientific) at a flow rate of 10 μLmin^-1^ of 0.1% formic acid in 10% aqueous solution of acetonitrile. After a 5-min desalting period the trap column was switched to analytical Acclaim PepMap RSLC C18 column (0.075 × 150 mm, 2 μm, 100 A, Thermo Fisher Scientific) and separation was performed using a nanocapillary Dionex Ultimate 3000 UHPLC system (Thermo Fisher Scientific) at a flow rate of 0.5 μL/min under the following gradient conditions: 10–20% B in 1 min, 20–60% B in 29 min, 60–95% B in 5 min, where solvent A was 0.1% formic acid, 2.5% acetonitrile and 2.5% isopropanol in water and solvent B was 0.08% formic acid in 90% acetonitrile and 5% isopropanol. The analytical column was connected directly to a solariX XR FT-ICR mass spectrometer (Bruker Daltonics) equipped with a 12 T superconducting magnet using an electrospray ion source. The instrument was calibrated externally using HP tune mix (Agilent Technologies) resulting in a typical mass accuracy below 2 ppm. Data acquisition and data processing were performed using ftmsControl 2.0, Hystar 4.0 and DataAnalysis 4.2 (Bruker Daltonics), respectively. Data were interpreted manually and only assignments having mass error below 3 ppm were considered as positive hits where the increments of the phosphopantetheine cofactor (molecular formula—C_11_H_21_N_2_O_6_P_1_S_1_) and PPL (molecular formula—C_8_H_13_N_1_O_1_) were 340.086 Da and 139.1 Da, respectively.

### Bioinformatic tools

Homologous sequences to both lincomycin and celesticetin genes were searched by BLAST (http://blast.ncbi.nlm.nih.gov/Blast.cgi). The BLASTX and BLASTP were used also for prediction of putative functions of encoded proteins in combination with Conserved Domains Database at http://www.ncbi.nlm.nih.gov/cdd [22].

## Results

### Comparative analysis of lincomycin and celesticetin biosynthetic gene clusters—search for candidate subunits of LS

In order to enable comparative analysis, the biosynthetic gene cluster for the other natural lincosamide antibiotic, celesticetin, was sequenced and deposited in GenBank (GQ844764); the detailed data are summarized in S1 Document. Both lincomycin and celesticetin biosynthetic gene clusters (Fig. 1) are localized differently in respective genomes (S1 Document) and contain specific putative resistance genes, three in lincomycin and one in celesticetin gene cluster (Table 2).

**Table 2:** Comparative analysis of lincomycin and celesticetin biosynthetic gene clusters.

LIN	CEL	Protein function assignment	Biosynthetic step assignment	Mutual identity (%)
*lmrA*	-	(S) MFS transporter	resistance	
*lmrB*	-	(S) 23S rRNA methyltransferase	resistance	
*lmrC*	-	(S) ABC transporter	resistance	
-	*ccr1*	(S) 23S rRNA methyltransferase	resistance	
*lmbA*	-	(S) Gamma-glutamyl transferase	PPL	
*lmbB1*	-	(C) L-DOPA-2,3-dioxygenase [25]	PPL	
*lmbB2*	-	(C) L-tyrosine 3-hydroxylase [26]	PPL	
*lmbW*	-	(S) C-methyltransferase	PPL	
*lmbX*	-	(S) DAP epimerase superfamily	PPL	
*lmbY*	-	(S) F-420 dependent reductase	PPL	
*lmbG*	-	(S) Methyltransferase	AS methylation	
*lmbU*	-	(C) Positive regulator [27,28]	regulation	
-	*ccb1*	(S) Acyltransferase	salicylate	
-	*ccb2*	(S) Salicyl-AMP ligase	salicylate	
-	*ccb3*	(S) Salicylate synthase	salicylate	
-	*ccb4*	(S) O-methyltransferase	AS methylation	
-	*ccb5*	(S) Dehydrogenase	salicylate	
*lmbC*	*ccbC*	(C) Adenylation domain [4]	condensation	54
***lmbD***	*ccbD*	(S) Unknown, no similarity	condensation	55
***lmbE***	*ccbE*	(S) Amidase, mycothiol conjugate	condensation	60
***lmbF***	*ccbF*	(S) Aminotransferase/racemase	condensation	39
***lmbIH***	*ccbIH*	(S) PmbA_TldD superfamily	regulation	63
*lmbJ*	*ccbJ*	(C) N-Methyltransferase [11,12]	N-methylation	60
*lmbK*	*ccbK*	(C) C8 phosphatase [13]	AS	63
*lmbL*	*ccbL*	(P) C8 dehydrogenase	AS	63
*lmbM*	*ccbM*	(P) C8 epimerase	AS	73
lmbN*^)^	*ccbN*	(C) C8 isomerase [14]+ (S) CP*^)^	AS + condensation	71 + 68*^)^
*lmbZ*	ccbZ*^)^	(P) C8 oxidoreductase + (S) CP*^)^	AS + condensation	64 + 68*^)^
*lmbP*	*ccbP*	(P) C8 kinase	AS	59
*lmbO*	*ccbO*	(C) C8 guanylyltransferase [13]	AS	64
*lmbS*	*ccbS*	(P) C8 aminotransferase	AS	74
*lmbR*	*ccbR*	(C) Transaldolase [14]	AS	69
***lmbQ***	*ccbQ*	(S) PmbA_TldD superfamily	regulation[57]	44
***lmbT***	*ccbT*	(S) Glycosyltransferase	AS	66
***lmbV***	*ccbV*	(S) Isomerase (mycothiol metabolism)	condensation	56

The genes coding for proteins with already confirmed/published functions are marked (C), the functions of other encoded proteins were assigned either according to sequence analysis combined with the prediction (P) in literature [14], or based on BLAST analysis only (S). The genes in bold were selected for further testing. For shared homologous genes (below the bold line) the percentage of mutual identity of encoded proteins is shown, for details see S1 Document. C8—octose; LIN—lincomycin; CEL—celesticetin;

*^)^—the relevant function, biosynthetic step assignment and sequence identity value concern only the marked gene; MFS—major facilitator superfamily; salicylate—both biosynthesis and attachment of the salicylate unit; AS—biosynthesis of amino sugar; PPL—biosynthesis of PPL

**Fig 2:**
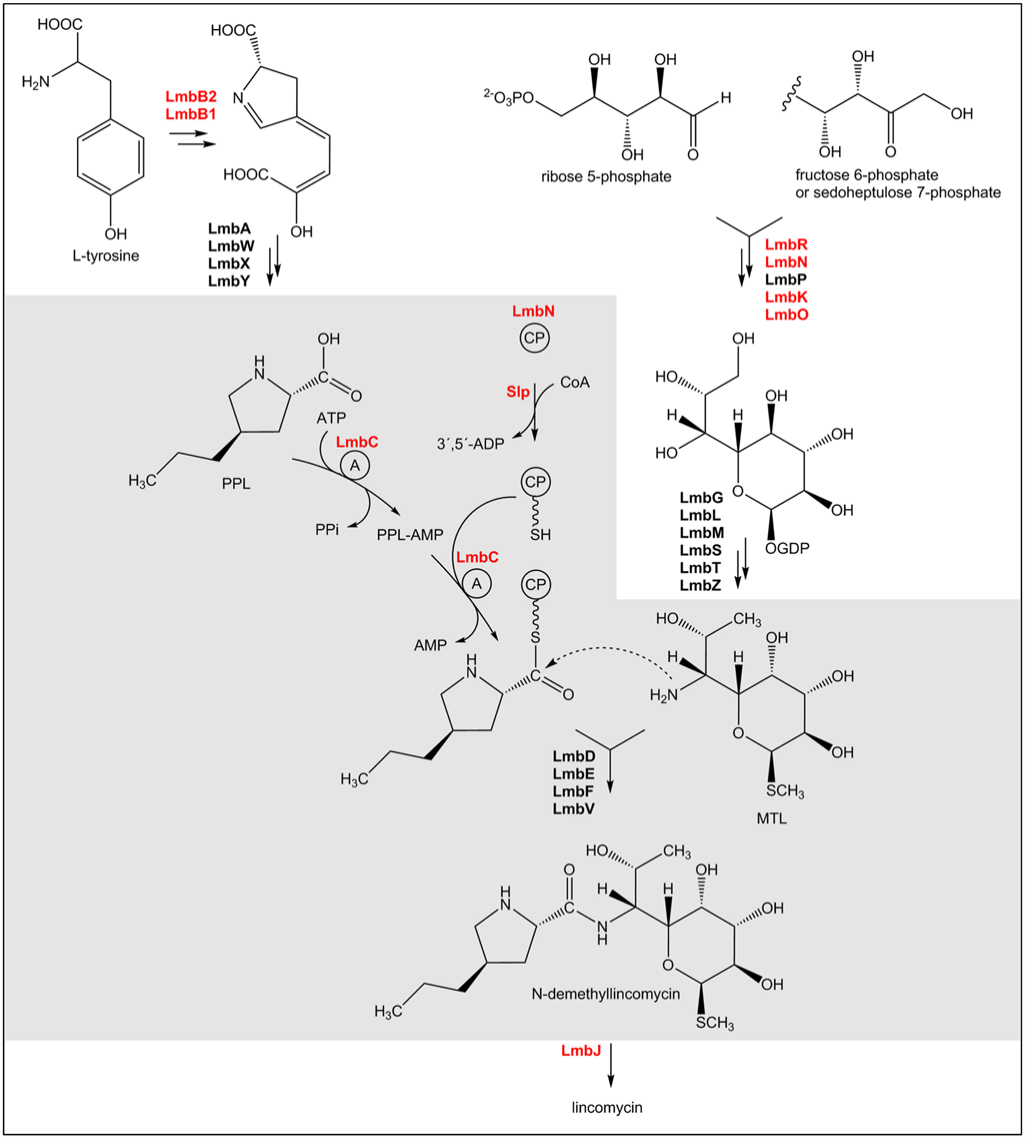
The lincomycin biosynthetic pathway. Gray background highlights the condensation step solved herein. Red—proteins with already proved functions, A—adenylation domain, CP—carrier protein, PPL—4-propyl-L-proline, MTL—methylthiolincosamide.

The comparative analysis of putative biosynthetic genes shows the prevalence of shared homologous gene pairs (a total of eighteen pairs, Table 2), while only eight lincomycin and five celesticetin specific genes were determined. Out of the lincomycin specific genes, a set of six genes (Fig. 2) was clearly assigned to PPL biosynthesis [6,18,23,24], two of them were already functionally proved [25,26], another specific gene, *lmbG*, codes for the putative methyltransferase corresponding to lincomycin specific S-methylation and *lmbU* is homologous to gene *novE* from novobiocin biosynthesis with proved regulatory function [27]. Moreover, the *lmbU* regulation function was confirmed later by the gene inactivation in a lincomycin producing strain [28]. Based on BLAST analysis, four out of five celesticetin specific genes putatively code for salicylate unit biosynthesis, activation and reductive attachment of activated salicylate unit to lincosamide core structure via a two-carbon linker: Encoded proteins Ccb3 and Ccb2 exhibit similarity to salicylate synthases [29,30] and salicyl-AMP ligase [31], respectively. Ccb1 shows similarity to O-acyltransferases including those exhibiting condensation activity in biosynthesis of peptide antibiotics [32]. Ccb5 contains a sequence motif shared by NADP dependent dehydrogenases indicating a function in the reduction step during the formation of the two-carbon linker. The last celesticetin specific gene *ccb4* encodes putative methyltransferase expected to catalyze the specific methylation of the amino sugar unit. The detailed analysis of celesticetin specific genes is summarized in S1 Document.

In summary, all the compound-specific genes code for clearly predictable activities corresponding to structural differences of the two lincosamides. In other words, the subunit composition of the lincomycin and celesticetin synthetases should be identical.

Putative NDLS subunits were searched among 18 Lmb proteins exhibiting homology with celesticetin ones (Fig. 1). First, the LmbJ, LmbK, LmbN, LmbO, and LmbR proteins (marked (C) in Table 2) with already proven functions in other than a condensation step can be excluded. Five other proteins (LmbL, LmbM, LmbZ, LmbP and LmbS; marked (P) in Table 2) exhibiting similarity to enzymes of sugar metabolism were assigned the functions in MTL biosynthesis predicted in [14]. LmbT protein exhibits only low homology to any protein of known function, but its C-terminal part exhibits similarity to glycosyltransferase domains indicating also possible participation in amino sugar biosynthesis. Nevertheless, the glycosyltransferase function in NDLS cannot be excluded.

From the remaining seven proteins, LmbC has recently been proved to function in NDLS. The candidates for so far unknown NDLS subunits should be sought among six Lmb proteins left (LmbD, LmbE, LmbF, LmbIH, LmbQ and LmbV). The prediction of their function based on sequence homology is impossible or at least ambiguous. The respective coding genes were thus inactivated and mutant strains further tested. Moreover, another two genes, previously assigned to sugar metabolism, were also inactivated and tested: *lmbT* (due to the uncertain function assignment mentioned above) and *lmbN* (explained in the next paragraph).

### Identification of sequence coding for putative CP domain of LS

Although the LmbN was recently proved to function as octulose 8-phosphate isomerase [14], a detailed gene cluster comparison revealed the putative CP coding sequence as part of *lmbN*. Surprisingly, the homologous putative CP coding sequence in celesticetin biosynthetic gene cluster is fused to another putative amino sugar biosynthetic gene, *ccbZ* (Fig. 1, zoomed).

The respective sequence codes for a ~ 80 amino acid residues long protein domain homologous to the peptidyl carrier protein family containing the phosphopantetheine binding site. The CP coding sequence forms the 5´-terminal part of the gene *lmbN* in the lincomycin cluster but a 3´-terminal part of the *ccbZ*, the adjacent gene of the *lmbN* counterpart in the celesticetin cluster. LmbN thus seems to be a bifunctional protein with two putative domains acting in different parts of lincomycin biosynthesis: the C-terminal domain in MTL biosynthesis, and the N-terminal domain in the condensation reaction. Three inactivation mutants were therefore prepared and tested in case of *lmbN*: in addition to full-length *lmbN* gene deletion (Δ*lmbN*) also *lmbN_*Δ*CP* with inactivated 5´- terminal part of the *lmbN* gene coding for putative CP domain, and *lmbN_*Δ*ID* with inactivated 3´- terminal part of the *lmbN* gene coding for isomerase domain acting in MTL biosynthesis.

### Testing of lmbN and lmbT inactivation mutant strains

**Fig 3:**
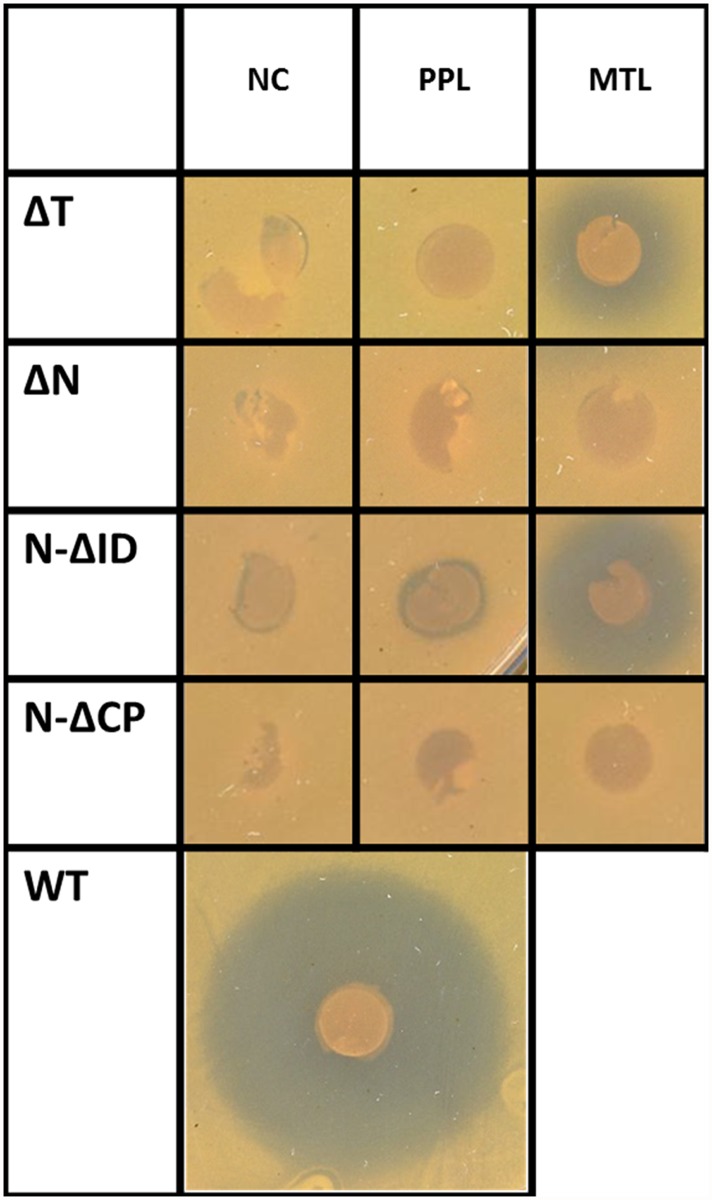
The biological activity assay of *S. lincolnensis* inactivation mutants. The inhibition zones correspond to the antibiotic production. WT—wild type strain (positive control); ΔT, ΔN, N-ΔID, N-ΔCP indicate the respective disrupted lincomycin biosynthetic gene or its part; NC—not complemented; PPL or MTL—complemented by the respective intermediate of lincomycin biosynthesis. For each sample, at least two independent cultivations were evaluated in triplicates as described in Material and methods. A typical result is shown.

All three tested *lmbN* inactivation mutant strains exhibited no inhibition zone in biological assay indicating disrupted biosynthesis of lincomycin (Fig. 3). Chemical complementation with PPL or MTL precursors restored the antibiotic production only in case of *lmbN_*Δ*ID* mutant with MTL added, confirming the published LmbN function in MTL biosynthesis. No inhibition zone formed by Δ*lmbN* and *lmbN_*Δ*CP* mutant complemented with any of NDLS substrates confirmed the predicted role of LmbN CP domain in the condensation reaction.

The *lmbT* gene inactivation disrupted the lincomycin production (Fig. 3). The antibiotic production was restored, similarly to *lmbN_*Δ*ID* mutant, by complementation with MTL, indicating participation of LmbT in MTL biosynthesis.

### Testing of inactivation mutant strains of remaining NDLS candidate genes

**Fig 4:**
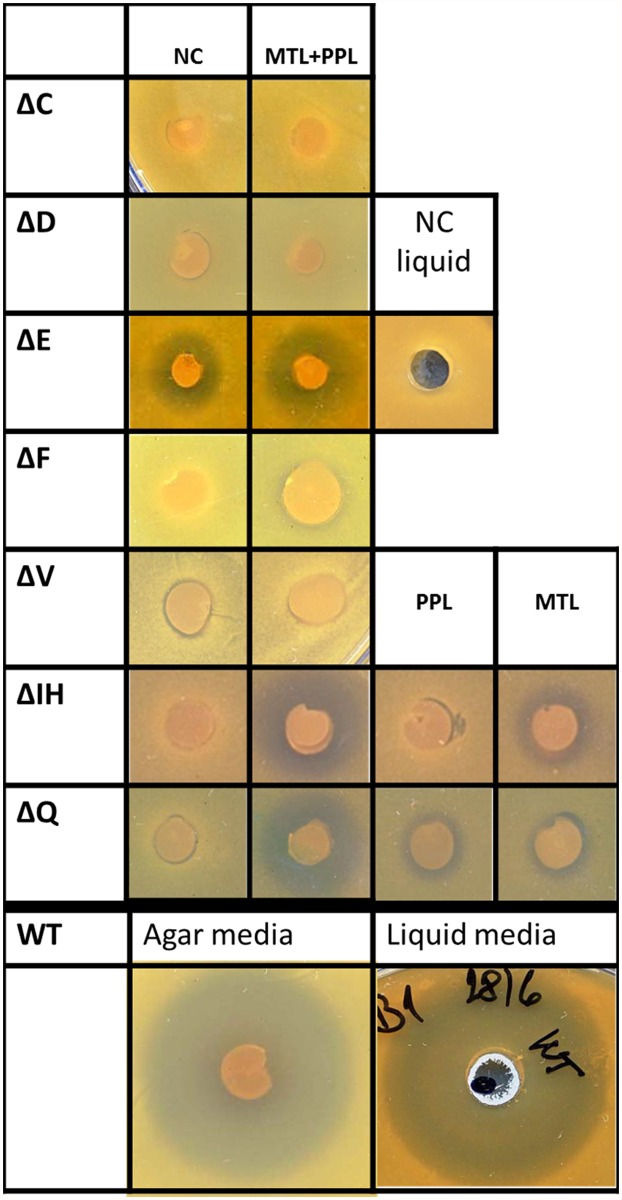
The biological activity assay of *S. lincolnensis* inactivation mutants. The inhibition zones correspond to the antibiotic production. WT—wild type strain (positive control); ΔC, ΔD, ΔE, ΔF, ΔV, ΔIH, ΔQ—indicate the respective disrupted lincomycin biosynthetic gene; NC—not complemented; PPL, MTL or PPL+MTL—indicate complementation by the respective intermediate of lincomycin biosynthesis or their combination. For each sample, at least two independent cultivations were evaluated in triplicates as described in Material and methods. A typical result is shown.

The mutant strains Δ*lmbC*, Δ*lmbD*, Δ*lmbE*, Δ*lmbF*, Δ*lmbIH*, Δ*lmbQ* and Δ*lmbV* with inactivated appropriate NDLS candidate genes, were tested in a biological activity assay (Fig. 4). The inactivation profiles of three mutant strains (Δ*lmbD*, Δ*lmbF* and Δ*lmbV*) resembled the profile of Δ*lmbC*, the mutant blocked in NDLS A-domain coding gene. The fully disrupted production of lincomycin, which cannot be restored by chemical complementation with a combination of both MTL and PPL precursors, indicates the participation of respective proteins in a condensation reaction together with already proven LmbC.

By contrast in case of mutant strains Δ*lmbIH* and Δ*lmbQ* the initially disrupted lincomycin production was restored by complementation with combined MTL and PPL precursors suggesting, that the respective proteins are not directly involved in the condensation reaction. Complementation with individual precursors, MTL or PPL, yielded clearly reduced inhibition zones when compared to complementation with both precursors, indicating some overall supporting or regulation function of respective LmbIH and LmbQ proteins in the lincomycin biosynthesis rather than direct catalytic function in the biosynthesis of any of its precursors.

In the mutant strain Δ*lmbE*, the lincomycin production was reduced, but not fully abolished, but the chemical complementation with both MTL and PPL combined didn’t change the size of the inhibition zone suggesting participation of LmbE in the condensation reaction. There are two possible interpretations of this result: either LmbE could participate in the condensation reaction, but its activity is not essential, or there is another activity encoded in the genome of producing strain, which is able to partially complement LmbE function. The later possibility seems to be more probable considering the available published data (see Discussion) together with the result of additional biological activity assay carried out in liquid cultivation media (Fig. 4). When the Δ*lmbE* strain was cultivated in liquid GYM media instead of GYM agar prior to the bioassay, no inhibition zone was formed on the testing plate while in case of the wild type *S. lincolnensis* strain (WT) the change of cultivation conditions didn’t affect the size of the inhibition zone. This result suggests the complementation by some gene with expression profile different from that of lincomycin biosynthetic genes.

### LC-MS analysis of fermentation media

The production of lincomycin and accumulation of both MTL and PPL precursors in fermentation media of mutant strains Δ*lmbC*, Δ*lmbD*, Δ*lmbE*, Δ*lmbF*, Δ*lmbN*, *lmbN_*Δ*ID*, *lmbN_*Δ*CP*, and Δ*lmbV* was determined after 60 and 120 hours of cultivation and compared with that of WT. We confirmed the fully abolished production of lincomycin in all tested mutant strains except for Δ*lmbE*. However, even in this case only a trace lincomycin production (~ 1 ‰ of WT production) was observed. For the MTL precursor, no remarkable difference in accumulation was observed in fermentation media of all tested mutant strains. All measured values were uniform and similar to that of WT, not exceeding a concentration of 0.5 μgmL^-1^ for both measured times of cultivation. A similar pattern was observed also for PPL when one of four neighboring genes *lmbC*, *lmbD*, *lmbE* or *lmbF* was inactivated. The accumulation of PPL in fermentation media of these mutant strains (all measured values varied from the limit of detection to the order of tens of ngmL^-1^) was even lower when compared to that of WT (hundreds of ngmL^-1^). Altogether these results indicate feedback regulation of the biosynthetic pathways of individual precursors.

The mutant strains Δ*lmbV* and *lmbN_*Δ*CP* exhibit increased PPL accumulation. Remarkably high PPL accumulation was observed in fermentation media of the Δ*lmbV* mutant. Measured concentrations of PPL (12.0 ± 1.6 μgmL^-1^) correspond with hypothetical production of 30.9 ± 4.2 μgmL^-1^ lincomycin, i.e. a typical value observed in wild type production strain. This indicates full lack of feedback regulation of PPL precursor biosynthesis in Δ*lmbV* mutant strain. Increased PPL accumulation was observed also for *lmbN_*Δ*CP* mutant strain, measured concentration of the precursor varied between values found in fermentation media of wild type strain and Δ*lmbV* mutant strain while when the MTL biosynthesis was disrupted (Δ*lmbN* and *lmbN_*Δ*ID*) the PPL accumulation resembled that of WT strain

### Analysis of *lmbN* gene expression, evidence of two forms of LmbN

**Fig 5:**
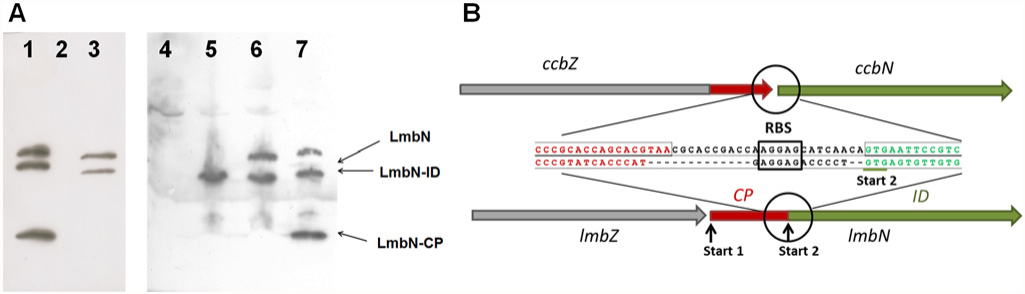
Evidence of two translation starts in LmbN coding gene. (A) Western blot analysis of LmbN forms produced by *S. lincolnensis*, and heterologously in *S. coelicolor*. 1 and 7: standards of MW (His_8_-tagged LmbN, His_8_-tagged LmbN-ID, His_8_-tagged LmbN-CP); 2: *S. lincolnensis* Δ*lmbN* mutant; 3: *S. lincolnensis* WT; 4: *S. coelicolor* M145 WT; 5: *S. coelicolor* M145 containing mutant form of *lmbN* with artificial stop codon introduced immediately downstream of the translation start 1; 6: *S. coelicolor* M145 containing native *lmbN gene*. (B) The detailed scheme of internal translation start (start 2) in *lmbN*. The red colour corresponds to CP domain coding sequence; the green colour corresponds to amino sugar isomerase domain (ID) coding sequence. RBS—ribosome binding site. Start 1—regular translation start producing full length LmbN.

The surprising fusion of sequences coding for CP domain and the enzyme acting in MTL biosynthesis evokes a question whether the native LmbN functions as an intact bifunctional protein, or if it is posttranslationally processed to produce two independent functional domains, the CP and the ID ones. To solve this question, the western blot analysis with immunodetection using polyclonal anti-LmbN antibody was carried out with the cell lysate of *S. lincolnensis* wild type strain (Fig. 5A, lane 3). Two immunoreactive proteins were detected. The recombinant His-tagged LmbN, stand-alone ID and CP were used as standards. The sizes of detected proteins correspond to full length LmbN and stand-alone ID standards when considering the size of His-tag (10 amino acid residues). However, no expected cleavage fragment of a size corresponding to CP was detected, making the hypothetical posttranslational LmbN cleavage questionable.

The alternative explanation of the existence of two LmbN forms can be deduced from detailed comparative analysis of *lmbN-lmbZ/ccbN-ccbZ* regions (Fig. 5B). In addition to the regular translation start of *lmbN* gene, the putative alternative ribosome binding site can be found in position corresponding to the ribosome binding site of homologous *ccbN* isomerase coding gene.

A putative translation product originating from this second translation start would give the stand-alone ID and thus corresponds in size to the smaller immunoreactive protein detected in wild type strain. To confirm the hypothesis of a second translation start, two variants of *lmbN* sequence were expressed in the heterologous host *S. coelicolor* M145. The first contained the intact *lmbN* sequence while in the second the *lmbN* translation start one was disrupted by a stop codon introduced immediately downstream of the regular start codon. The western blot analysis of appropriate cell lysates including *S. coelicolor* M145 as a negative control confirmed the existence of the second translation start in *lmbN* gene (Fig. 5A, lanes 4–6). No immunoreactive protein was detected in the host strain *S. coelicolor* M145. The same strain bearing native *lmbN* sequence provided two immunoreactive proteins corresponding in size to those detected in wild type strain, while the introduction of the construct with artificial stop codon following start one resulted in production of only a single protein, corresponding to stand-alone ID starting from translation start two.

### The evidence of CP domain function

**Heterologous production and purification of CP domain**. To test the CP function in NDLS we made an attempt to produce a full length LmbN protein with N-terminal His-tag in *E. coli*. However, the purified recombinant LmbN protein aggregated. As the stand-alone CP was previously confirmed to keep its function in NDLS (see the results of inactivation experiments for *lmbN_*Δ*ID* in Fig. 3), the *E. coli* expression system for heterologous production of stand-alone LmbN-CP with C-terminal His-tag was developed. The soluble protein was obtained and purified by affinity chromatography. The LC-MS analysis of the purified recombinant CP demonstrated that the prevailing form corresponds in molecular mass to *apo*-CP, *i.e*. the CP lacking covalently bound 4′-phosphopantetheine (Table 3), the obligatory cofactor of CPs. The *apo*-CP was ~11 times more abundant than the *holo*-form. Conversion of *apo*-CPs to the *holo*-form is catalyzed by phosphopantetheinyl transferases (PPTases). It was described previously [33–36] that PPTases operating in secondary metabolism are usually different from those acting in primary metabolism. The *E. coli* host strain is deficient in the broad specificity secondary metabolism PPTase of Sfp-type [37] which could efficiently convert the *apo*-LmbN-CP to the *holo*-form. To yield *holo*-forms of NRPS-CPs produced heterogously in *E. coli*, additional broad specificity PPTases of Sfp type were used [33,35,38–40].

**Table 3:** MS analysis of LmbN-CP.

	*apo*-CP [M+H]/ abundance	*holo*-CP [M+H]/ abundance	*holo*-CP acetylated [M+H]/ abundance	*holo*-CP-PPL [M+H]/ abundance
**CP produced without Slp**	9688.00 a.m.u. / 100%	10028.10 a.m.u. / 9%	N.o.	N.A.
**CP co-produced with Slp(*in vivo* phosphopantetheinylation)**	9687.99 a.m.u. / 100%	10028.08 a.m.u. / 23%	10070.09 a.m.u. / 35%	N.A.
**CP co-produced with Slp: acylation reaction with PPL**	9687.99 a.m.u. / 59%	N.o.	10070.09 a.m.u. / 100%	10167.17 a.m.u. / 46%
**CP produced without Slp(*in vitro* phosphopantetheinylation)**	9687.99 a.m.u. / 100%	10028.08 a.m.u. / 58%	N.o.	N.A.
**CP produced without Slp (*in vitro* phosphopantetheinylation and acylation)**	9687.98 a.m.u. / 63%	10028.08 a.m.u. / 5%	N.o.	11167.17 a.m.u. / 100%
**Theoretical [M+H]**	9687.97 a.m.u.	10028.06 a.m.u.	10070.07 a.m.u.	10167.16 a.m.u.

The mass corresponds to the mature LmbN-CP form. Percent values display abundance in comparison to the most abundant form of LmbN-CP in the sample. a.m.u.—atomic mass unit, N.o.—Not observed, N.A.—not applicable.

**Identification and heterologous production of S. lincolnensis PPTase**. As no gene encoding PPTase was identified in the lincomycin gene cluster, the whole *S. lincolnensis* genome was searched for a gene encoding PPTase of broad specificity. We modified the approach developed for the identification of promiscuous Sfp-type PPTase Svp from *S. verticillus* [35] (for detailed procedure see S2 Document) and obtained the sequence coding for a protein (named Slp) consisting of 226 amino acid residues, homologous to broad specificity PPTase of Sfp-type SCO6673-like (75.8% identity). The sequence of *slp* gene was deposited in GenBank under the accession number KM252689. C-terminally His-tagged Slp was heterologously produced in *E. coli* and purified by affinity chromatography.

Phosphopantetheinylation of LmbN-CP domain by the Slp was tested by both, the *in vitro* reaction as well as the co-expression of both proteins *in vivo* in *E. coli*. The LC-MS analysis revealed partial, but significant conversion of *apo*-CP to the *holo*-form in both reaction systems (Table 3). *The holo*-form reached nearly 60% of the *apo*-form content using *in vitro* reaction. A lower yield of *holo*-CP form was obtained *in vivo*, where *apo*-CP was ~4 times more abundant than *holo*-CP. Nevertheless, the production of additional form, acetylated *holo*-CP, was observed in this system. The total content of *holo*-CP and acetylated *holo*-CP produced *in vivo* was similar to the abundance of *holo*-CP form observed *in vitro*, i.e. nearly 60% of the *apo*-form content.

**The functional testing of CP**. Finally, we tested whether LmbC, the NDLS A-domain, transfers activated PPL precursor to *holo*-CP of LmbN. Two parallel reactions were carried out with LmbN-CP processed using *in vitro* or *in vivo* phosphopantetheinylation system. An expected product with molecular mass 10167.17 Da corresponding to the complex of *holo*-CP with bound PPL was demonstrated by LC-MS in both parallel reactions (Table 3). The conversion of *holo*-CP into *holo*-CP-PPL complex was in both cases almost complete. This provides an evidence that the A-domain LmbC together with LmbN-*holo*-CP activate the PPL precursor of lincomycin. C-terminal ID of LmbN is thus not essential for attachment of PPL onto the LmbN-CP domain.

It should be noted that mass spectrometry data reveal the presence of additional not fully mature forms of LmbN-CP (starting from methionine or formyl-methionine). Both additional LmbN-CP forms follow the trend of mature LmbN-CP protein.

## Discussion

We demonstrate the hybrid nature of LS, the key enzyme in the biosynthesis of lincomycin and related celesticetin. In the quaternary structure, the LS combines some components of typical NRPSs (A-domain and CP), reflecting the incorporation of an amino acid precursor, with highly specific protein activities associated to amino sugar attachment via the formation of amide bond. These unusual proteins were assigned to LS activity based on the results of a series of inactivation experiments. With the exception of LmbE/CcbE homologous pair they exhibit only very low (LmbF/CcbF, LmbV/CcbV) or even no (LmbD/CcbD) sequence homology when compared to all database available proteins either with already confirmed functions or just putative proteins mined from thousands of genome projects.

### LS components resembling NRPS initiation module

The NRPS-like component of LS is, besides the already published stand-alone A-domain LmbC/CcbC [4], the newly identified CP. The NRPS CPs are usually fused to functionally relevant domains, most often A-domains and condensation domains [5]. One could thus reasonably assume that the fusion partner of CP domain identified in the lincosamide biosynthesis might be a protein with function analogous to the absent condensation domain, e.g. the LS subunit involved in the amino sugar precursor attachment. However, the results clearly exclude such a possibility for several reasons: 1. The CP is fused to mutually nonhomologous proteins in both biosyntheses, LmbN in lincomycin but CcbZ in celesticetin biosynthesis. 2. Both the CP fusion partners act in the biosynthesis of an amino sugar precursor, however both in well-defined biosynthetic steps far apart from the condensation. In case of LmbN the C8 isomerase function was already confirmed [14], the C8 oxidoreductase function of CcbZ is fairly predictable. 3. Neither isomerase, nor the oxidoreductase could have any obvious functional relationship with the condensation reaction. The connection of CP with these fusion partners thus seems to be rather coincidental. Generally the fusion of CP with proteins of nonrelated functions is rare, but such arrangements have already been described in the literature [41,42].

The additional identified protein indirectly involved in the NDLS function of lincomycin biosynthesis is Slp from *S. lincolnensis*, a PPTase *converting* the *apo*-LmbN-CP to the active *holo*-form with a phosphopantetheine cofactor bound. Generally the PPTases involved in secondary metabolism can be encoded either directly in the respective biosynthetic gene cluster [33,43,44] or somewhere else in the genome [35,45,46] as is the case of Slp. According to the sequence homology, the identified Slp protein belongs to F/KES subfamily of Sfp-type PPTases [47]. Regarding the gene context (S2 Document) and fair efficiency in LmbN-CP activation, Slp could be a broad substrate specificity PPTase of *S. lincolnensis*. Results of experiments with recombinant proteins completed the functional characterization of NDLS subunits exhibiting similarity to components of NRPS systems. LmbC and LmbN-CP form together a functional block resembling NRPS initiation module for PPL activation.

### Putative LS components involved in an amino sugar unit attachment

The attachment of amino sugar to the α-carboxyl group of an amino acid is relatively rare in secondary metabolites. One specialized system was described in the biosynthesis of streptothricin [8] where the amino sugar is attached solely using NRPS components. The amide bond is formed by a condensation domain of unusual NRPS using the CP-bound amino acid or peptide intermediate. Another less clarified NRPS dependent system participates in the biosynthesis of nourseothricin [42]. In several other already described biosynthetic pathways the connection of an amino acid and an amino sugar via an amide bond is generally catalyzed by a single specialized enzyme without participation of any NRPS-like component. This is for example the case of cysteinyl-tRNA synthetase homologue in the biosynthesis of mycothiol and similar bacillithiol [48–51]. In puromycin biosynthesis the amide bond is formed by a tyrosinyl-aminonucleoside synthetase by a NRPS-independent mechanism [52]. To elucidate the mechanism of condensation of precursors in lincosamide biosynthesis, no overall analogy with any already described biosynthetic system could thus be used as an inspiration.

When considering the remaining four putative NDLS subunits LmbD, LmbE, LmbF and LmbV, the bioinformatic analysis revealed some remarkable sequence homology only for LmbE protein. In its entire length LmbE exhibits significant homology with two proteins from the metabolism of mycothiol, the major thiol in most actinomycetes, which replaces glutathione function and acts as a central compound of a group specific detoxification system [53]. LmbE exhibits 32.4% sequence identity to functionally characterized mycothiol-S-conjugate amidase Mca [49] (O53430, E = 2e-32), that cleaves the amide bond between the amino sugar and amino acid moieties in various S-conjugates of mycothiol during the mycothiol recycling. The second protein from mycothiol metabolism, homologous to LmbE, is MshB [49] (30.8% identity, P9WJN3, E = 2e-22) that cleaves off the acetyl residue bound via amide bond to the amino sugar in mycothiol biosynthesis. This could support the hypothesis of LmbE being the NDLS subunit catalyzing the formation of the amide bond between PPL and MTL lincomycin precursors. However, Mca and MshB catalyze the reaction in the reverse direction, i.e. the cleavage of the amide bond, not its formation. Moreover, as mentioned above, the L-cystein entering the biosynthesis of mycothiol is not activated using A-domain and CP.

Another argument supporting the notion that LmbE/CcbE is truly a LS subunit is provided by the organization of genes in the two compared lincosamide biosynthetic gene clusters. The genes *lmbC*,*D*,*E*,*F/ccbC*,*D*,*E*,*F* form a compact group, a subcluster within the whole biosynthetic gene cluster (Fig. 1). This subcluster is localized in the two clusters in a different gene neighborhood, but the internal order of the genes in the subcluster is identical. This compact arrangement suggests the functional relationship of relevant encoded proteins. Regarding the already confirmed function of LmbC/CcbC, also the remaining trio of proteins is supposed to participate in the condensation reaction. Almost identical are also the production profiles of relevant inactivation mutant strains *ΔlmbC*, *ΔlmbD*, *ΔlmbE* and *ΔlmbF*: 1. The lincomycin production cannot be restored by feeding with a combination of both PPL and MTL precursors (Fig. 4) 2. None of the precursors is accumulated in the fermentation media of inactivation mutants. The PPL accumulation is even lower when compared to the wild type strain indicating a stringent negative control resulting from a block in the condensation step. The encoded proteins (C,D,E,F) thus probably form a core of respective LS regulated as a whole. A partial lincomycin production of mutant strain *ΔlmbE* under specific cultivation conditions can be sufficiently explained by substitution activity of homologous protein(s) from mycothiol metabolism.

The prediction of LmbD/CcbD protein function based on sequence homology completely fails. The presence of a pair of conserved ORFs in both compared biosynthetic gene clusters itself is the only evidence that it is an authentic existing protein. Besides their high mutual homology (identity: 57%; E-value: 5e-98) no other similarity to any database available sequence can be found even to any short conserved motif enabling the prediction of at least a type of catalytic activity. The LmbD/CcbD protein thus seems to be a unique product arising specifically from evolution of lincosamide biosynthesis. A supporting indication of the authenticity could be also the fact that the recombinant LmbD protein can be prepared by overexpression in *E. coli*, partially even in a soluble form. The purified LmbD, however, exhibits a strong tendency to form aggregates or high molecular weight oligomers. In view of the absence of any sequence motif suggesting any enzymatic function and regarding the properties of the recombinant protein one can speculate that the LmbD/CcbD protein could function as a central structural element of multimeric LS.

The LmbF/CcbF protein exhibits, according to the Conserved Domain Database [22], similarity to aspartate aminotransferase protein family (E-value below 1e-10) [54] covering only a short region indicating another, however related function. Enzymes of this protein family are known to make use of pyridoxal phosphate cofactor interacting with an amino group of an amino acid to form a Schiff base (aldimine) intermediate which could be a substrate, besides transamination, also for racemization or other types of modifying reactions. LmbF/CcbF thus probably modifies the conformation of the amino acid precursor to the form suitable for condensation reaction. Alternatively, and even more probably, it could “only” fix the precursor in a proper LS site and in a suitable orientation for subsequent reaction. The low mutual identity of the LmbF/CcbF protein pair (Table 2) could reflect a different recognized amino acid (PPL vs L-proline).

The last hypothetical component of LS could be, based on the results of inactivation experiments, the LmbV/CcbV protein. The respective coding gene is, similarly to *lmbN*, localized outside the CDEF “core” subcluster. The production profile of Δ*lmbV* mutant (higher accumulation of PPL) contrasts starkly with those of the putative core LS proteins C, D, E and F discussed above, but is similar to *lmbN_*Δ*CP*, i.e. the strain lacking one of already confirmed NDLS components. The prediction of LmbV/CcbV function based on the sequence homology is rather difficult as it exhibits only a low homology to any protein of known function. However, its N-terminal part exhibits low but significant similarity to proteins of DinB_2 superfamily. Proteins of this superfamily often belong to isomerases including those participating in sugar metabolism. Remarkably, the DinB_2 superfamily includes mycothiol S-transferase [55] and also another functionally characterized protein from mycothiol metabolism, the mycothiol maleylpyruvate isomerase (MDMPI) N-terminal domain (2NSG_A) [56]. The MDMPI acts in mycothiol dependent assimilation of aromatic compounds catalyzing the conversion of maleylpyruvate to fumarylpyruvate. It is difficult to identify a sequence homology between LmbV and MDMPI but the pairwise alignment reveals three amino acid residues (His^52^, Glu^144^ and His^148^) forming zinc binding site conserved also in LmbV. LmbV thus probably is a zinc-binding protein with a function of the type resembling that of MDMPI or mycothiol S-transferase in mycothiol metabolism, even though it is hard to suspect the direct evolution linkage.

The overall hypothetic conception of LS structural arrangement could be, based on current knowledge, summarized as a hybrid multimeric system consisting of two functional blocks representing evolutionary totally different concepts of condensing systems. The first one, functionally fully elucidated for the lincomycin biosynthesis, consists of a stand-alone A-domain (LmbC) and CP domain (LmbN-CP) resembling a complete NRPS initiation module, activates PPL, the lincomycin amino acid precursor. The latter one, NRPS-dissimilar, catalyzing the amino sugar unit attachment, exhibits some features of mycothiol conjugates condensing system. Most probably, the central catalytic activity of the amino sugar attachment, i.e. the formation of the amide bond, is driven by the LmbE protein, homologous with Mca and MshB amidases from mycothiol metabolism. LmbF and LmbV can assist in this attachment step by the interaction with the amino acid and the amino sugar units, respectively (fixing and/or modifying them). LmbD could form the structural core of the whole LS or its NRPS-dissimilar block. It is not clear if the similarity of LmbV and, in particular, LmbE to proteins from mycothiol metabolism reflects just an “evolutionary signature” referring to a common evolutionary origin or rather the direct involvement of mycothiol or its conjugates in the lincomycin biosynthesis.

### Proteins with ambiguous function in lincosamide biosynthesis

Additionally there are two homologous protein pairs common for lincomycin and celesticetin biosynthesis—LmbIH/CcbIH and LmbQ/CcbQ, whose functions have not yet been solved. The results published about LmbQ [57] suggest its regulatory function in lincomycin biosynthesis. Moreover, both LmbQ and LmbIH proteins belong to the same protein family (PmbA_TldD superfamily; [58]) even though their mutual sequence homology is very low. Genes coding for proteins of this family occur in bacterial genomes quite often, usually as homologous pairs localized in a close neighborhood. The TldD/TldE proteins in *E. coli* possess a proteolytic activity involved in the maturation of specific peptides [59]. The PmbA_TldD superfamily protein pairs have already been identified also in actinobacteria in biosynthetic gene clusters of secondary metabolites [60]. Regarding the evidence in *E. coli*, our initial hypothesis was that LmbIH could posttranslationally cleave the bifunctional LmbN protein and produce the matured CP and ID, thus performing a regulatory function. The experiments with *S. lincolnensis* producing strain did not confirm this hypothesis, however a regulatory function is reasonably supposed (S1 Document).

### Evolutionary aspects of lincosamide antibiotic biosynthesis

From an overall perspective of evolution of lincosamide antibiotics biosynthesis, a valuable knowledge was achieved on the mobility of CP coding sequence between two neighboring genes in the cluster. First, it documents an interesting principle, which contributes to the evolution, namely the mobility of an element on the subgene level. Second, it represents another supporting argument indicating the direction of evolution in the biosynthesis of lincosamides. The preserved internal start of translation in fusion *lmbN* gene indicates, that the CP coding sequence originally formed part of another gene and that its fusion with the ID coding part of *lmbN* occurred relatively recently. The gene arrangement conserved in celesticetin biosynthetic gene cluster, where CP coding sequence forms part of *ccbZ* without autonomous translation start, probably corresponds to an evolutionarily older state. This is already the second argument pointing to the evolutionary direction from proline-incorporating lincosamides to PPL-incorporating ones. In a recent paper [4] we documented that A-domain recognizing specifically the unusual precursor PPL evolved from a protein family of originally proline-specific A-domains.

The establishment of a multimeric LS, an absolutely unique hybrid system performing condensation of amino acid and amino sugar units, represents the first milestone in the evolution of lincosamide biosynthesis. The incorporation of an unusual PPL amino acid precursor into the structure of an antibiotic was the second breakpoint in the evolution as it gave rise to a remarkably more efficient variant of lincosamide antibiotic, lincomycin. The primary prerequisite of this evolutionary step was the acceptance of genes encoding the biosynthesis of unusual PPL precursor into the lincosamide biosynthetic gene cluster followed by substrate specificity adaptation of the LS A-domain [4] but without any change in the overall LS subunit composition. Accordingly, the six relevant genes (*lmbA*, *B*, *B2*, *W*, *X* and *Y*) are present in the lincomycin biosynthetic gene cluster but not in the celesticetin one. Their highly homologous counterparts in the biosynthesis of some pyrrolobenzodiazepines [18,23,24,61] and hormaomycin [62] indicate both the origin and the mode of the gene acceptance by the lincomycin biosynthetic gene cluster (the horizontal gene transfer).

The comparison of the two lincosamide biosynthetic gene clusters represents together with described experimental results an impressive record of evolution of a unique hybrid system for the condensation of two basic precursors of lincosamide antibiotics as well as of its qualitative shift to the more efficient natural product lincomycin. The tools utilized by nature in this process are based on shifting and fusions of variably complex genetic units (gene subclusters, individual genes as well as gene segments) both within a single biosynthetic gene cluster as well as among various clusters by horizontal gene transfer. This overall gene cluster evolution history was combined with the modification of substrate specificity of the central catalytic activity of an evolving biosynthetic pathway, the driving force of this process being the evolutionary selection pressure for production of a more efficient natural compound. This is a particular illustration of general principles of evolution of secondary metabolites, but also an inspiration for a rational design of hybrid biologically active compounds.

## Supporting Information

S1 Document Isolation and sequence characterization of celesticetin biosynthetic gene cluster. (PDF)

S2 Document Identification and expression of Slp, a broad specificity PPTase from *S. lincolnensis*. (PDF)

S1 Table Primers. (PDF)

S2 Table LC-MS analysis of lincomycin and precursors. (PDF)

S3 Table Production of recombinant LmbN protein forms. (PDF)
