# Supplementary material for: Lincosamide Synthetase—A Unique Condensation System Combining Elements of Nonribosomal Peptide Synthetase and Mycothiol Metabolism
Source: PLoS One. 2015 Mar 5;10(3):e0118850. doi: 10.1371/journal.pone.0118850 (PMC4351081; doi:10.1371/journal.pone.0118850)
Supplement: S1 Document — (PDF) [file pone.0118850.s001.pdf]

Document S1 – Isolation and sequence characterization of celesticetin biosynthetic gene cluster.

Isolation and sequencing of celesticetin biosynthetic gene cluster

The type strain *Streptomyces caelestis* ATCC 15084 producing celesticetin was used as a source of chromosomal DNA for cosmid library construction. The probes for library screening were designed according to sequences of predicted homologous *S. lincolnensis* ATCC 25466 genes *lmbJ*, *lmbM*, *lmbS* and *lmbZ.* Two overlapping cosmids CK1 and CK2 covering the complete celesticetin gene cluster sequence were obtained. A 36543 bp long DNA region bearing the whole celesticetin cluster was sequenced and deposited in GenBank under accession number GQ844764.

Location of the celesticetin gene cluster in the genome

The cluster of celesticetin biosynthetic and resistance genes spans a region of 27350 bp and contains 24 putative genes. The boundaries of the celesticetin biosynthetic cluster were deduced by comparative genomics. Genes *sca1* and *sca2* flanking the celesticetin gene cluster (Figure DS1-1) show significant similarity to *S. coelicolor* primary metabolism genes *sco4741* and *sco2677* (followed by *sca3* and *sca4* genes homologous to *sco2676* and *sco2675*) and were therefore assigned to the primary metabolism.

Figure DS1-1. Location of the celesticetin gene cluster in the genome and the layout of the cosmids CK1 and CK2.

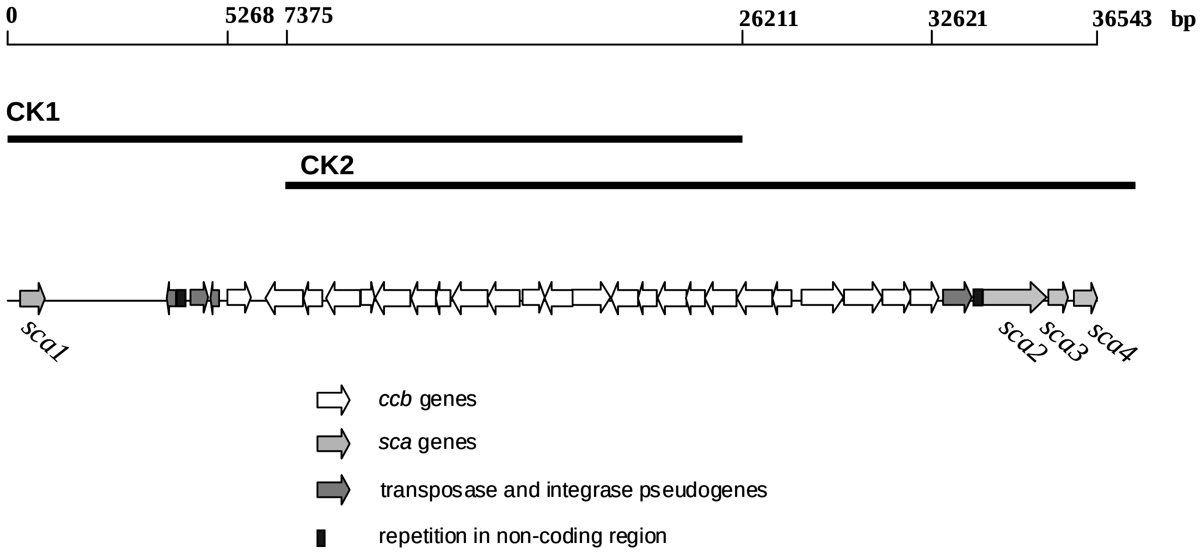

The top line denotes the *S. caelestis* chromosome. Lines underneath denote the cosmids overlapping the gene cluster. ORFs of the cluster and its vicinity are indicated as arrows, repetitions in non-coding region are indicated as black boxes. The repetitions, integrase and transposase pseudogenes indicate the cluster positioning in the *S. caelestis* genome.

ccb – celesticetin biosynthetic genes

sca – *S.caelestis* genes of primary metabolism with homologous counterparts in *S. coelicolor* genome

Moreover, in the intergenic region between the *sca1* gene (homologous to *sco4741*) and the putative celesticetin resistance gene *ccr1,* a 112 bp long sequence was found showing 81% identity with intergenic region between genes *sco2678* and *sco2677* of the *S. coelicolor* A3(2) genome (position 194502-194615). Another 215 bp long homologous sequence with 85% identity to the same region (position 194444-194658) was detected in the intergenic region between ccb5 and sca2 genes, i.e. at the other border of the antibiotic cluster. Mutual identity of these two repetitions is 94 %. Both repetitions are accompanied by transposase and/or integrase pseudogenes, other elements of the integration machinery. All these findings specified the mechanism and integration site of the celesticetin biosynthetic cluster within the host genome.
Summarized the location of the celesticetin and related lincomycin biosynthetic cluster in the genomic contexts is not equivalent. The lincomycin cluster is placed between genes homologous to *S. coelicolor* sco4638 and sco4612, while the celesticetin one lies between the genes homologous to *S. coelicolor* sco2677 a sco2678.

Sequence analysis of celesticetin biosynthetic gene cluster

The functions of putative proteins encoded by celesticetin biosynthetic cluster genes were assigned based on BLAST similarity and comparative analysis with the lincomycin biosynthetic gene cluster. The degree of identity with proteins from lincomycin biosynthesis is shown (Table DS1-1) as well as the closest homologues corresponding to the first match (the lowest E-value) according to TBLASTN analysis except the lincomycin biosynthetic gene cluster. The lincomycin and celesticetin biosynthetic gene clusters share18 homologous genes mostly coding for proteins involved in the amino sugar biosynthesis or assigned to the condensation reaction. The identity of their protein products varies from 39 to 73 percent. The functional assignment of lincomycin/celesticetin homologous genes is discussed in the main text. Additionally, another five specific genes with putative biosynthetic function (*ccb1-ccb5*) and one specific putative resistance gene *ccr1* were identified in the celesticetin biosynthetic gene cluster.

Resistance gene

Gene *ccr1* is the only putative resistance gene in the celesticetin biosynthetic cluster. Its protein product Ccr1 belongs, similarly to the putative resistance protein LmrB from lincomycin biosynthesis, to the family of 23S rRNA methyltransferases. Nevertheless, Ccr1 and LmrB are not directly evolutionarily linked. The fact that the compared lincosamide clusters do not share any resistance genes indicates higher frequency of horizontal transfer of individual genes coding for antibiotic resistance if compared to HGT of functional blocks of biosynthetic genes.

Table DS1-1. *Sequence analysis of celesticetine biosynthetic gene cluster*

Gene	Protein length	Biosynthetic step assignment	Protein function or sequence homology *)	Closest homologue according to BLAST<br>• Homologous protein<br>• Organism (GenBank acc. No.)<br>• Identities (% of identity)	
				S. lincolnensis	Other organisms
ccr1	341	resistance	23S rRNA methyltransferase		TlrD<br>*Streptomyces fradiae* (X97721)<br>137/256 (54%)
ccbIH	484	regulation	PmbA_TldD superfamily	LmbIH<br>(ABX00605)<br>296/464 (63%)	peptidase U62 modulator of DNA gyrase<br>*Dictyoglomus turgidum* DSM 6724 (ACK41515)<br>177/476 (37%)
ccbJ	256	N-methylation	(C) N-methyltransferase [5,6]	LmbJ<br>(ABX00606)<br>152/251 (60%)	putative methyltransferase<br>*Saccharothrix espanaensis* DSM 44229 (CCH31873)<br>103/252 (41%)
ccb1	437	salicylate	Acyltransferase (salicylate attachment)		Acyltransferase<br>*Mycobacterium kansasii* ATCC 12478 (AGZ53527)<br>67/194 (35%)
ccbK	185	amino sugar	(P) C8 phosphatase	LmbK<br>(ABX00607)<br>110/173 (63%)	D-alpha,beta-D-heptose 1,7-bisphosphate phosphatase<br>*Solibacter usitatus* Ellin6076 (ABJ81431)<br>85/172 (49%)
ccbL	436	amino sugar	(P) C8 dehydrogenase	LmbL<br>(ABX00608)<br>270/426 (63%)	nucleotide sugar dehydrogenase<br>*Thermodesulfatator indicus* DSM 15286 (AEH45078)<br>143/432 (33%)
ccbM	325	amino sugar	(P) C8 epimerase	LmbM<br>(ABX00609)<br>239/323 (73%)	UDP-glucose 4-epimerase<br>*Methanosarcina mazei* strain Goe1 (AAM30830)<br>145/318 (46%)
ccbN	202	amino sugar	(P) C8 isomerase	LmbN<br>(ABX00610)<br>143/201 (71%)	sugar phosphatase<br>*Sorangium cellulosum* 'So ce 56' (CAN93908)<br>76/197 (39%)

ccbZ	427	N-terminal domain: amino sugar	(P) C8 oxidoreductase	LmbZ (ABX00611) 213/332 (64%)	putative dehydrogenase uncultured *Acidobacteria bacterium* (AAP58526.1) 128/324 (40%)
		C-terminal domain: condensation	(P) CP (L-proline transfer)	LmbN (ABX00610) 51/75 (68%)	KALB_2180, putative acyl carrier protein *Kutzneria albida* DSM 43870 (AHH95549.1) 25/54 (46%)
ccbF	416	condensation	aminotransferase	LmbF (ABX00603) 158/397 (39%)	class I and II aminotransferase *Actinoplanes* sp. N902-109 (AGL19165) 111/352 (32%)
ccbE	274	condensation	amidase	LmbE (ABX00602) 164/273 (60%)	putative mycothiol conjugate amidase *Ilumatobacter coccineus* YM16-304 DNA (BAN01850) 101/292 (35%)
ccbD	355	condensation	unknown function	LmbD (ABX0060) 199/356 (55%)	no significant similarity
ccbC	505	condensation	(C) adenylation domain activation of L-proline [7]	LmbC (ABX00600) 278/506 (54%)	amino acid adenylation domain *Anabaena* sp. 37 (JF803645) 194/544 (36%)
ccbP	323	amino sugar	(P) C8 kinase	LmbP (ABX00612) 189/320 (59%)	predicted kinase related to galactokinase *Sorangium cellulosum* So0157-2 (AGP37028) 121/329 (37%)
ccbO	232	amino sugar	(P) C8 guanylyltransferase	LmbO (ABX00613) 144/222 (64%)	nucleotidyl transferase family protein *Acidobacterium capsulatum* ATCC 51196 (ACO34012) 86/219 (38%)
ccbS	386	amino sugar	(P) C8 aminotransferase	LmbS (ABX00614) 273/367 (74%)	glutamine-scyllo-inositol transaminase *Syntrophobotulus glycolicus* DSM 8271 (ADY57269) 179/384 (47%)
ccbR	222	amino sugar	(P) transaldolase	LmbR (ABX00615) 151/218 (69%)	putative transaldolase *Cellulomonas flavigena* DSM 20109 (ADG76474) 100/211 (47%)
ccbQ	385	regulation	PmbA_TldD superfamily	LmbQ (ABX00616)	putative modulator of DNA gyrase *Actinoplanes friuliensis* DSM 7358 (AGZ46015)

				164/377 (44%)	75/236 (32%)
ccbT	435	amino sugar	glycosyltransferase	LmbT (ABX00617) 283/435 (66%)	putative glycosyltransferase *Yersinia enterocolitica* W22703 (CBX73549) 107/418 (26%)
ccbV	237	condensation	isomerase	LmbV (ABX00618) 133/238 (56%)	putative mycothiol maleylpyruvate isomerase N-TD *Thermobispora bispora* DSM 43833 (ADG90196) 92/219 (42%)
ccb2	548	salicylate	(P) salicyl-AMP ligase		SsfL1, putative salicyl-CoA ligase *Streptomyces sp.* SF2575 (ADE34495) 309/543 (57%)
ccb3	455	salicylate	(P) salicylate synthase		putative anthranilate synthase component I *Saccharopolyspora erythraea* (CAM01971) 244/431 (57%)
ccb4	369	O-methylation	O-methyltransferase		O-methyltransferase family 2 protein *Pirellula staleyi* DSM 6068 (ADB16222) 133/347 (38%)
ccb5	351	salicylate	dehydrogenase		putative dehydrogenase *Streptomyces bingchenggensis* BCW-1(ADI09787) 217/331 (66%)

*) protein functions already confirmed (C) or predicted (P), in other cases (unmarked) the predicted type of reaction based on sequence homology only. The length of encoded proteins is expressed as a number of amino acid residues. C8 – octose; salicylate – both biosynthesis and attachment of the salicylate unit; condensation – reactions leading to formation of amide bond between the amino sugar and the amino acid precursors. Identities – number of identical residues / number of compared residues according to TBLASTN.

Celesticetin specific biosynthetic genes

Genes *ccb1-ccb5* have no counterparts in the lincomycin biosynthetic gene cluster and, except for the gene *ccb4*, they putatively participate in the biosynthesis and attachment of celesticetin specific salicylate unit. Putative protein Ccb3 exhibits 43% similarity with the salicylate synthase MbtI of *Mycobacterium tuberculosis.* MbtI is capable of both ring isomerization and pyruvate lyase activity and catalyzes direct conversion of chorismate to salicylate [1]. The similarity of Ccb3 with MbtI or Irp9 indicates that in the biosynthesis of celesticetin salicylate unit the chorismate is directly converted to salicylate in the same way as in siderophore biosynthesis of *Mycobacterium tuberculosis* [1] and *Yersinia enterocolitica* [2]. The Ccb2 protein, exhibiting 57% identity to putative salicyl-AMP ligase SdgA of *Streptomyces sp.* WA46 [3] is supposed to catalyze the activation of salicyl-SCoA. Ccb5 is similar to many NADP-dependent alcohol dehydrogenases indicating that this enzyme catalyzes the reduction step during the formation of the two-carbon chain, which connects TCA and salicylate. The gene *ccb1* is localized apart from the genes coding for salicylate unit biosynthesis. Its protein product Ccb1 contains putative condensation domain found in several multi-domain enzymes which synthesize peptide antibiotics [4]. In the celesticetin biosynthesis, the Ccb1 putatively catalyzes the attachment of the salicylate unit via the two-carbon chain to the amino sugar unit. The remaining celesticetin specific gene *ccb4* codes for putative O-methyltransferase required for methylation of the celesticetin amino sugar unit in position 7´.

Genes shared by celesticetin and lincomycin biosyntheses with ambiguous function

There are three homologous gene pairs common for lincomycin and celesticetin biosynthesis - *lmbIH*/*ccbIH*, *lmbQ*/*ccbQ* and *lmbT*/*ccbT*. Functions of respective encoded proteins have not yet been solved. The inactivation experiments however showed, that these proteins cannot relate directly to the condensation reaction as the disrupted production of antibiotic by the respective mutant strains was restored by feeding the cells with one or both precursors. The results of inactivation experiments assigned LmbT to the MTL biosynthesis, which corresponds well with the presence of a glycosyltransferase sequence motif in *lmbT*. It is not apparent, in which MTL biosynthetic step it should participate, because the proposed remaining steps of MTL biosynthesis do not presume glycosyltransferase activity [8].

The results published about LmbQ [9] suggest its regulatory function in lincomycin biosynthesis. This fully corresponds with our results: Inactivation of *lmbQ* gene abolished the antibiotic production, but the production pattern related to feeding with individual precursors or the combination of both MTL and PPL does not allow the assignment of LmbQ either to the biosynthesis of any precursor, or to the condensation step. Identical results were obtained also when the gene coding for LmbIH was inactivated. Similarly to LmbQ, also LmbIH will probably be involved in some regulation or will have an overall supporting function in lincomycin biosynthesis. As was shown in our previous work [10], both LmbQ and LmbIH proteins belong to the same protein family (PmbA_TldD superfamily) even though their mutual sequence homology is very low. Genes coding for proteins of this family are not obligatorily present in all bacterial genomes but they occur quite often, usually as pairs. The two relevant coding sequences are frequently localized in a close neighborhood. They have already been identified also in biosynthetic gene clusters of secondary metabolites. The TldD/TldE proteins in *E. coli* possess a proteolytic activity involved in the maturation of

gyrase inhibitor microcin B17 and in degradation of CcdA/CcdA41 antidotes participating in ccd poison-antidote system of the F plasmid [11], while the functions of similar protein pairs found in actinobacteria, e.g. CalR6/CalR5 in the biosynthesis of calicheamicin in *Micromonospora echinospora*, remain unknown [12]. Regarding the evidence in *E. coli*, our initial hypothesis was that LmbIH could posttranslationally cleave the bifunctional LmbN protein and produce the matured CP and ID, thus performing a regulatory function. However, the experiments with *S. lincolnensis* producing strain did not confirm this hypothesis.

References:

1. Harrison AJ, Yu M, Gårdenborg T, Middleditch M, Ramsay RJ, et al. (2006) The structure of MbtI from Mycobacterium tuberculosis, the first enzyme in the biosynthesis of the siderophore mycobactin, reveals it to be a salicylate synthase. J Bacteriol 188: 6081-6091.
2. Kerbarh O, Ciulli A, Howard NI, Abell C (2005) Salicylate biosynthesis: overexpression, purification, and characterization of Irp9, a bifunctional salicylate synthase from Yersinia enterocolitica. J Bacteriol 187: 5061-5066.
3. Ishiyama D, Vujaklija D, Davies J (2004) Novel pathway of salicylate degradation by Streptomyces sp. strain WA46. Applied and environmental microbiology 70: 1297-1306.
4. Stachelhaus T, Mootz HD, Bergendahl V, Marahiel MA (1998) Peptide bond formation in nonribosomal peptide biosynthesis catalytic role of the condensation domain. J Biol Chem 273: 22773-22781.
5. Najmanova L, Kutejova E, Kadlec J, Polan M, Olsovska J, et al. (2013) Characterization of N-Demethyllincosamide Methyltransferases LmbJ and CcbJ. Chembiochem 14: 2259-2262.
6. Bauer J, Ondrovicova G, Najmanova L, Pevala V, Kamenik Z, et al. (2014) Structure and possible mechanism of the CcbJ methyltransferase from Streptomyces caelestis. Acta Crystallogr D Biol Crystallogr 70: 943-957.
7. Kadlcik S, Kucera T, Chalupska D, Gazak R, Koberska M, et al. (2013) Adaptation of an L-Proline Adenylation Domain to Use 4-Propyl-L-Proline in the Evolution of Lincosamide Biosynthesis. PLoS One 8(12) doi:10.1371/journal.pone.0084902.
8. Sasaki E, Lin CI, Lin KY, Liu HW (2012) Construction of the Octose 8-Phosphate Intermediate in Lincomycin A Biosynthesis: Characterization of the Reactions Catalyzed by LmbR and LmbN. J Am Chem Soc 134: 17432-17435.
9. Zengliang W, Qunfei Z, Shuhong G, Changhua C, Wen L (2010) Construction of the New Genetic Manipulation Method of Streptomyces lincolnensis and lmbQ Gene Function Verification. Biotechnology Bulletin 11: 038.
10. Janata J, Najmanova L, Novotna J, Hola K, Felsberg J, et al. (2001) Putative lmbI and lmbH genes form a single lmbIH ORF in Streptomyces lincolnensis type strain ATCC 25466. Antonie Van Leeuwenhoek 79: 277-284.
11. Allali N, Afif H, Couturier M, Van Melderen L (2002) The highly conserved TldD and TldE proteins of Escherichia coli are involved in microcin B17 processing and in CcdA degradation. J Bacteriol 184: 3224-3231.
12. Ahlert J, Shepard E, Lomovskaya N, Zazopoulos E, Staffa A, et al. (2002) The calicheamicin gene cluster and its iterative type I enediyne PKS. Science 297: 1173-1176.
