# Supplementary material for: Lincosamide Synthetase—A Unique Condensation System Combining Elements of Nonribosomal Peptide Synthetase and Mycothiol Metabolism
Source: PLoS One. 2015 Mar 5;10(3):e0118850. doi: 10.1371/journal.pone.0118850 (PMC4351081; doi:10.1371/journal.pone.0118850)
Supplement: S2 Document — (PDF) [file pone.0118850.s002.pdf]

Document S2

Identification and expression of Slp, a broad specificity PPTase from *S. lincolnensis*

Methods:

Isolation and identification of slp gene sequence from S. lincolnensis genome

Sequence fragment ~250 bp was PCR amplified from genomic DNA of *S. lincolnensis* using the degenerated primers P1 and P3-2 (Table S1) designed for identification of Svp, a *Streptomyces verticillus* PPTase of Sfp-type [1]. The PCR product was inserted into pGEM-T (Promega), sequenced and homology with PPTases was confirmed by nucleotide blast program at NCBI web site. The complete sequence of the *slp* gene was obtained by PCR using the degenerated primers slpup and slpdow designed according to conserved neighborhood of the PPTase encoding genes (Figure DS2-1). The PCR product ~1 kbp bearing *slp* and adjacent regions defined by the primers slpup and slpdow was inserted into pGEM-T and used for determination of the *slp* sequence.

Heterologous production of full-length and His-tagged Slp protein

The primers slpfor and slp1rev (Table S1) were used for the amplification of full-length sequence of *slp*. The PCR product was inserted into pACYCDuet-1 (Novagen) via the *Nde*I and *Xho*I restriction sites to give rise construct pslp1 for production of full-length Slp. The primers slpfor and slp2rev were used for the preparation of expression construct for producing C-terminally His_8-tagged Slp: The amplified sequence was inserted into pET42b vector (Novagen) via the *Nde*I and *Xho*I restriction sites to give rise construct pslp2. The proteins were produced in *E. coli* BL21(DE3) (Novagen), transformed by pslp1 or pslp2, as appropriate. LB medium with chloramphenicol (34 mgL^{-1}) or kanamycin (30 mgL^{-1}), respectively, was inoculated and grown at 37 °C. At OD_{600} = 0.7, the culture was cooled down to 27 °C and the overexpression was induced by 0.4 mM isopropyl-β-D-thiogalactopyranoside. The cells were grown for an additional 4 hours at 27 °C and harvested by centrifugation. Production of Slp was verified by SDS-PAGE.

Purification of His-tagged Slp

His_8-tagged Slp was purified from crude cell extracts prepared by ultrasonic homogenization in TS-8 buffer (20 mM Tris-HCl, 100 mM NaCl, pH 8.0), using HiTrap™ Chelating HP Columns (GE Healthcare). His-tagged proteins were eluted by TS-8 buffer with 250 mM imidazole. The purified protein was dialyzed overnight against 50 mM Tris-HCl (pH 8.8) and stored at 4°C.

Computational anylysis

Sequence of *slp* was identified and analyzed using blast search programs at the NCBI web site (http://blast.ncbi.nlm.nih.gov/Blast.cgi). Protein sequences of the Slp homologues were retrieved from GenBank. Sequence identities of Slp with the homologues were calculated in Geneious 5.5.6 [2] based on pairwise alignments generated using MAFFT software version 7.181 at the CBRC web site (http://mafft.cbrc.jp/alignment/server) [3].

Results and discussion:

Covalently bound phosphopantetheine cofactor converting *apo*-CPs of NRPSs to their *holo*-form is prerequisite of the CP functionality. The CPs can be poorly phosphopantetheinylated when overproduced heterologously in *E. coli* due to absence of appropriate PPTase catalysing the *apo*- to *holo*-form conversion. This problem is routinely overcome by using a PPTase of Sfp-type with broad substrate specificity, for example Sfp or Svp [1,4-6]. PPTases of this

class usually modify *apo*-CPs of NRPSs as well as PKSs, in contrast to AcpS-type PPTases that usually modify CPs of FAS in primary metabolism. Nevertheless, Sppt PPTase of the Sfp-type from *Synechocystis* sp. is specific for FAS CP modification [7].

Since LmbN-CP from lincomycin biosynthesis was heterologously produced almost only in *apo*-form, a PPTase of Sfp-type was essential for preparation of functional *holo*-LmbN-CP. We decided to find out the gene encoding such PPTase in lincomycin producing strain *S. lincolnensis*.

A PPTase involved in the biosynthesis of corresponding secondary metabolite can be encoded directly in the biosynthetic gene cluster of the metabolite [4,8,9] or somewhere else in the genome [1,10,11]. Published data concerning lincomycin gene cluster [12,13] do not mention a PPTase encoding gene. Indeed, tblastn analysis of the lincomycin cluster (EU124663) did not find a homologue of either Sfp-type PPTase (CAA44858) or AcpS-type PPTase (CAB12269). Therefore, gene coding for a PPTase phosphopantetheinylating LmbN-CP should be located outside of the cluster. However, this gene is not easily achievable since *S. lincolnensis* genome has not been sequenced yet.

To identify a gene encoding *S. lincolnensis* PPTase with broad substrate specificity we modified the approach that was developed in similar situation for identification of promiscuous Sfp-type PPTase Svp of *S. verticillus* [1]. Using of published degenerated primers [1] derived from conserved motifs P1 and P3 a PPTase, a DNA fragment of expected size ~250 bp was amplified from *S. lincolnensis* genomic DNA. Blastx search found the highest similarity of the amplified sequence with PPTases of *S. coelicolor* (SCO6673), *S. avermitilis* (SAV1748) and *S. verticillus* (Svp). Inspection of neighborhood surrounding the genes coding for these three PPTases revealed homology among sequences both upstream and downstream of the genes (Figure DS2-1). The proteins encoded upstream the PPTase genes (SCO6672, SAV1749, Orf1) are similar to a metallophosphatases (NCBI CDD cl13995). Genes downstream the PPTase genes encode mutually homologous proteins of unknown function (SCO6674 and SAV1747). This finding suggested that also a *S. lincolnensis* PPTase can be encoded in similar context. Therefore, to amplify an unknown gene of *S. lincolnensis* PPTase, a pair of degenerated primers slpup and slpdow was designed according to those neighboring sequences (Figure DS2-2). We expected a PCR product of the length at least ~800 bp defined by slpup and slpdow primers and including the sequence encoding entire PPTase. Indeed, the PCR resulted in ~1 kbp product which was sequenced and analyzed by blastx. The sequence contained an ORF coding for a protein consisting of 226 amino acid residues, a searched putative *S. lincolnensis* PPTase named Slp. Sequence of *slp* gene was deposited in GenBank under the accession number KM252689.

Slp corresponds to a Sfp-type of PPTases (~240 residues) but not to an AcpS-type of PPTases (~120 residues). According to the sequence similarity, Slp belongs to F/KES subfamily of Sfp-type PPTases [14] containing motif P1A (Figure DS2-3). Slp has high sequence identity with functionally described streptomycete PPTases of F/KES subfamily: SCO6673, SCO6673-like and Svp (Table DS2-1). SCO6673-like and Svp are considered as broad specific PPTases [1,11]. Functionally described non-streptomycetes PPTases of Sfp-type, PcpS, EntD and SppT [4,7,11], has a lower similarity to Slp (Table DS2-1). Low sequence identity was found also between Slp and promiscuous Sfp that belongs to W/KEA subfamily. Similarity of Slp with promiscuous PPTases SCO6673-like and Svp suggested Slp as suitable candidate for phosphopantetheinylation of *apo*-LmbN-CP.

The soluble full-length Slp as well as C-terminally His8-tagged Slp were produced in *E. coli*. The full-length Slp was co-produced with LmbN-CP and used for *in vivo* phosphopantetheinylation of the LmbN-CP. His_8-tagged Slp was purified in a one-step procedure to near homogeneity and immediately used for in *in vitro* phosphopantetheinylation of LmbN-CP. Typical yield was 0.5 mg of pure His_8-tagged Slp per 100 mL of cell culture.

The Slp migrates in SDS PAGE as a protein about of 32 kDa (calculated MW for native Slp is 24297). Also mobility of His-tagged Slp differs from the calculated MW value. (Figures DS2-4 and DS2-5). Similar discrepancy was described previously also for PPTases of Sfp-type, for example in case of Sfp and Svp [1,15].

The PPTases most similar to Slp have a similar gene context of their coding genes as Slp, in many cases. Recently, Bunet *et al.* [11] reported that in majority of the streptomycete sequenced genomes a gene coding for homologue of PPTase SCO6673 (homologue of Slp) is located downstream of a conserved gene encoding a SCO6672 homologue. These findings suggest that pair of degenerated primers slpup1 and slpup2 can serve for identification and amplification of a PPTase SCO6673 gene homologues from diverse streptomycetes genomes.

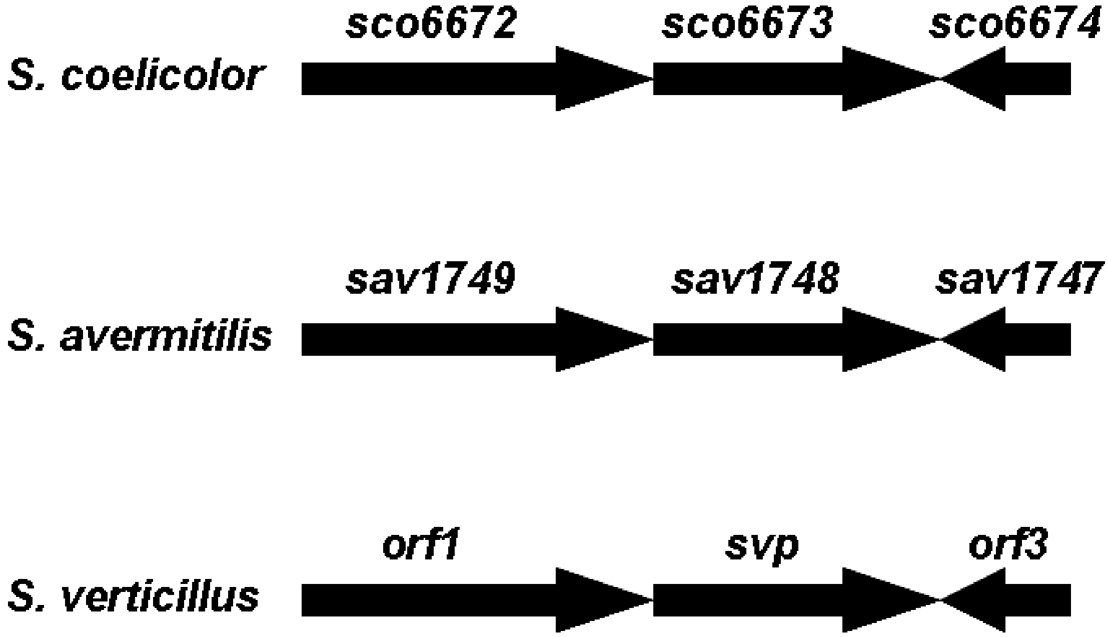

Figure DS2-1: Neighborhood of genes encoding PPTases SCO6673, SAV1748 and Svp. SCO6672, SAV1749, ORF1 are similar to a metallophosphatases. SCO6674, SAV1747, Orf3 - unknown function.

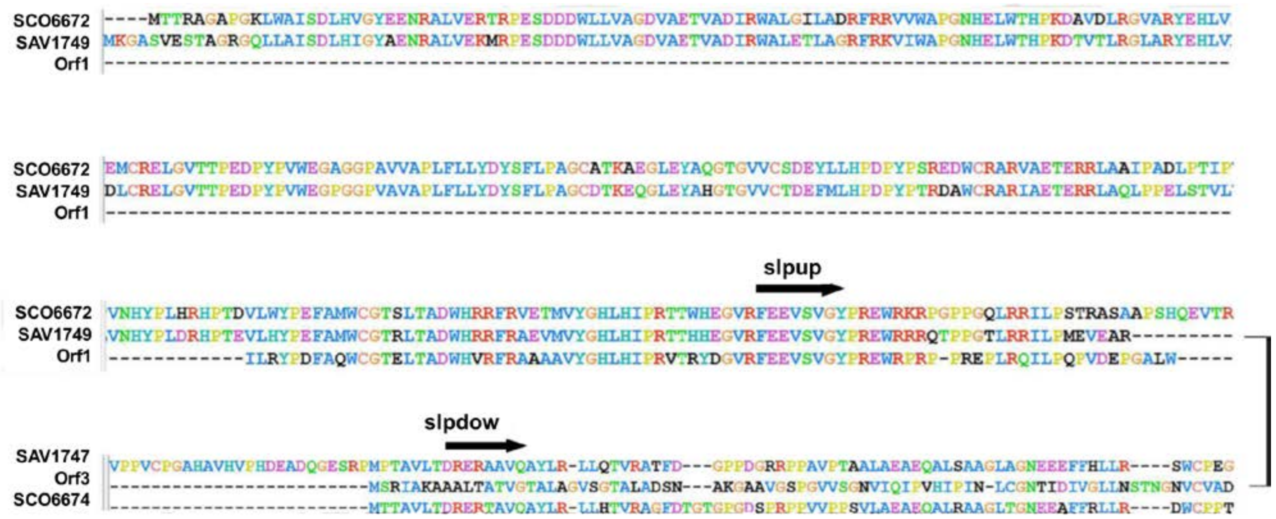

Figure DS2-2: Alignment of protein sequences of *S. avermitilis* (SAV1747, SAV1749), *S. coelicolor* (SCO6672, SCO6674) and *S. verticillus* (Orf1, Orf3). Genes encoding these proteins surround the genes encoding homologues of *slp* gene: PPTases SCO6673, SAV1748 and Svp. Position of corresponding PPTase encoding genes is marked with the broken line. Amino acid residues used for design of degenerated primers slpup and slpdow are marked by arrows.

Table DS2-1: Sequence identity of the Slp with functionally described PPTases of Sfp-type.

PPTase	GenBank No.	identity with Slp (%)
SCO6673 - like	[11]	75.8
SCO6673	CAA19952.1	71.4
Svp	AAG43513.1	60.7
PcpS	AAG04554.1	25.9
EntD	CAB57861.1	20.7
Sppt	BAA10326.1	16.6
Sfp	CAA44858.1	13.6

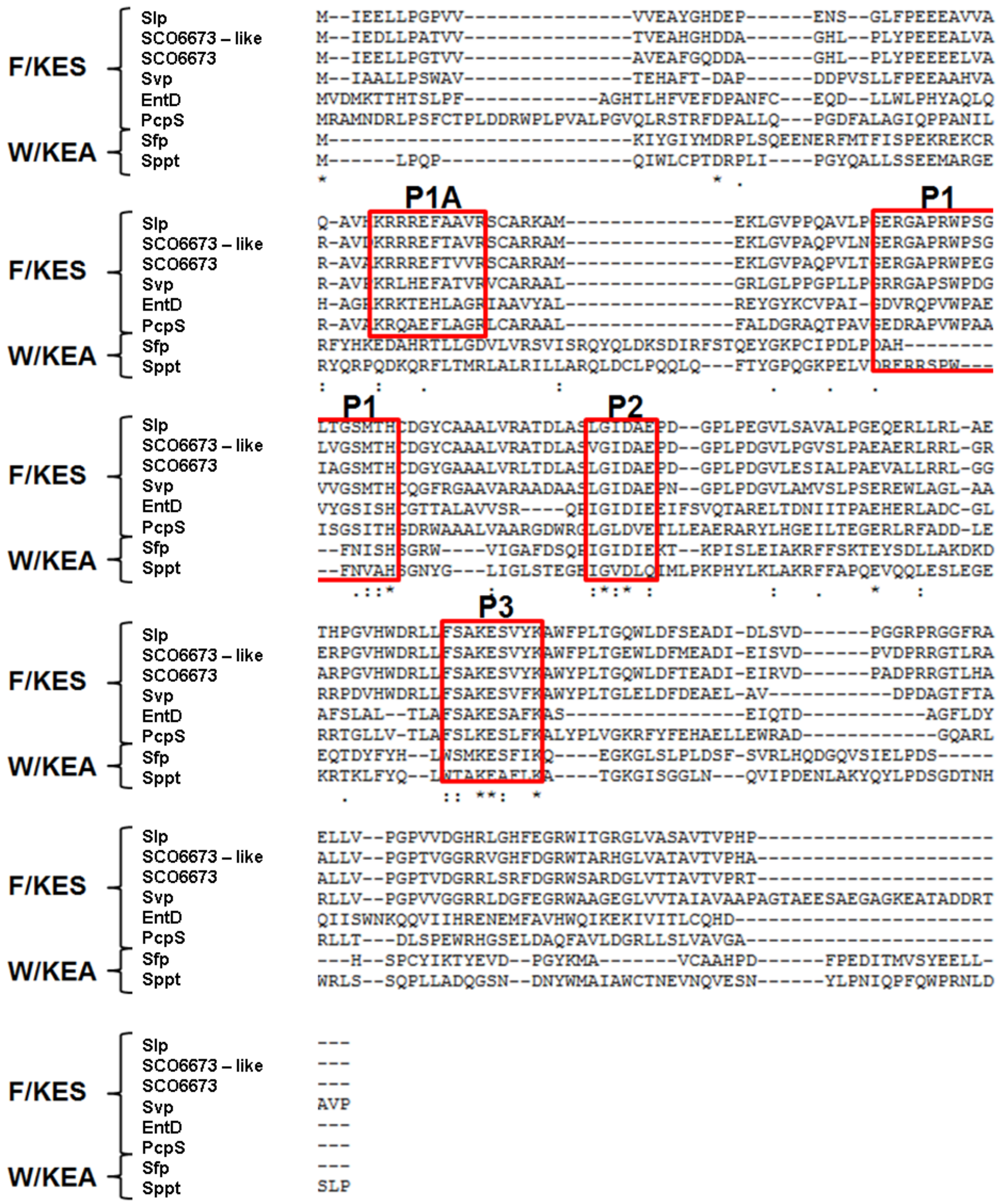

Figure DS2-3: Sequence alignment of the Slp with functionally described PPTases of Sfp-type. Proteins belonging to the F/KES and W/KEA subfamily are marked. Conserved motifs are in red boxes.

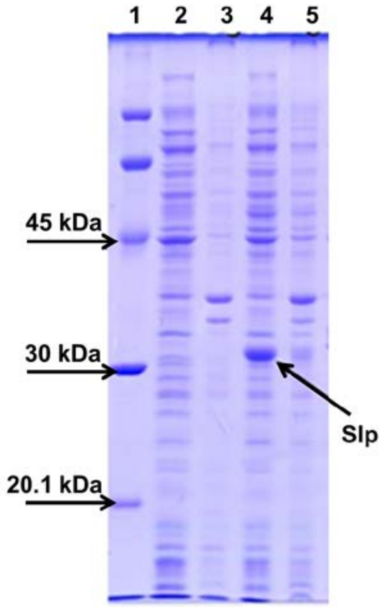

Figure DS2-4: Heterologous production of the full-length Slp in *E. coli*: SDS-PAGE analysis. Lane 1: MW standards, 2: soluble fraction of cells lysate before IPTG induction, 3: insoluble fraction of cells lysate before IPTG induction, 4: soluble fraction of cells lysate after IPTG induction, 5: insoluble fraction of cells lysate after IPTG induction. Note that the mobility of the full-length Slp does not correspond to the calculated MW value (24297).

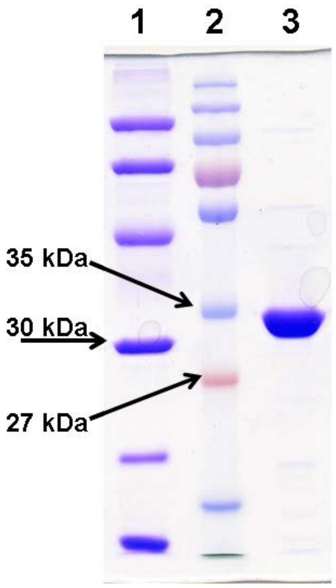

Figure DS2-5: Mobility of the His_8-tagged Slp in SDS-PAGE. Lanes 1 and 2: MW standards, 3: His_8-tagged Slp. Note that the mobility of the His_8-tagged Slp does not correspond to the calculated MW value.

References:

1. Sánchez C, Du L, Edwards DJ, Toney MD, Shen B (2001) Cloning and characterization of a phosphopantetheinyl transferase from *Streptomyces verticillus* ATCC15003, the producer of the hybrid peptide-polyketide antitumor drug bleomycin. Chem Biol 8: 725-738.
2. Geneious version 5.5.6 created by Biomatters. Available from http://www.geneious.com/
3. Katoh K, Standley DM (2013) MAFFT multiple sequence alignment software version 7: Improvements in performance and usability. Mol Biol Evol 30: 772-780.
4. Lambalot RH, Gehring AM, Flugel RS, Zuber P, LaCelle M, et al. (1996) A new enzyme superfamily-the phosphopantetheinyl transferases. Chem Biol 3: 923-936.
5. Garneau S, Dorrestein PC, Kelleher NL, Walsh CT (2005) Characterization of the formation of the pyrrole moiety during clorobiocin and coumermycin A_1 biosynthesis. Biochemistry 44: 2770-2780.
6. Méjean A, Mann S, Vassiliadis G, Lombard B, Loew D, et al. (2010) *In vitro* reconstitution of the first steps of anatoxin-a biosynthesis in *Oscillatoria* PCC 6506: From free L-proline to acyl carrier protein bound dehydroproline. Biochemistry 49: 103-113.
7. Roberts AA, Copp JN, Marahiel MA, Neilan BA (2009) The *Synechocystis* sp. PCC6803 Sfp-type phosphopantetheinyl transferase does not possess characteristic broad-range activity. Chembiochem 10: 1869-1877.
8. Harris AKP, Williamson NR, Slater H, Cox A, Abbasi S, et al. (2004) The *Serratia* gene cluster encoding biosynthesis of the red antibiotic, prodigiosin, shows species- and strain-dependent genome context variation. Microbiology-Sgm 150: 3547-3560.
9. Stanley AE, Walton LJ, Kourdi Zerikly M, Corre C, Challis GL (2006) Elucidation of the *Streptomyces coelicolor* pathway to 4-methoxy-2,2´-bipyrrole-5-carboxaldehyde, an intermediate in prodiginine biosynthesis. Chem Commun (Camb): 3981-3983.
10. Finking R, Solsbacher J, Konz D, Schobert M, Schäfer A, et al. (2002) Characterization of a new type of phosphopantetheinyl transferase for fatty acid and siderophore metabolism in *Pseudomonas aeruginosa*. J Biol Chem 277: 50293-50302.
11. Bunet R, Riclea R, Laureti L, Hôtel L, Paris C, et al. (2014) A single Sfp-type phosphopantetheinyl transferase plays a major role in the biosynthesis of PKS and NRPS derived metabolites in *Streptomyces ambofaciens* ATCC23877. PLoS ONE 9(1): e87607. doi:10.1371/journal.pone.0087607
12. Peschke U, Schmidt H, Zhang HZ, Piepersberg W (1995) Molecular characterization of the lincomycin-production gene cluster of *Streptomyces lincolnensis* 78-11. Mol Microbiol 16: 1137-1156.
13. Koberska M, Kopecky J, Olsovska J, Jelinkova M, Ulanova D, et al. (2008) Sequence analysis and heterologous expression of the lincomycin biosynthetic cluster of the type strain *Streptomyces lincolnensis* ATCC 25466. Folia Microbiol 53: 395-401.
14. Copp JN, Neilan BA (2006) The phosphopantetheinyl transferase superfamily: phylogenetic analysis and functional implications in cyanobacteria. Appl Environ Microbiol 72: 2298-2305.
15. Nakano MM, Corbell N, Besson J, Zuber P (1992) Isolation and characterization of *sfp*: a gene that functions in the production of the lipopeptide biosurfactant, surfactin, in *Bacillus subtilis*. Mol Gen Genet 232: 313-321.
