# Supplementary material for: Lincosamide Synthetase—A Unique Condensation System Combining Elements of Nonribosomal Peptide Synthetase and Mycothiol Metabolism
Source: PLoS One. 2015 Mar 5;10(3):e0118850. doi: 10.1371/journal.pone.0118850 (PMC4351081; doi:10.1371/journal.pone.0118850)
Supplement: S1 Table — (PDF) [file pone.0118850.s003.pdf]

TABLE S1. Primers

Primer	Sequence (5' to 3')	Inactivated gene
**A) Inactivation primers**		
dCf	TCCTTCATGTCGTCCTCCGTTCGACTCTCCACACTCCTCATTCCGGGGATCCGTCGACC	lmbC
dCr	TCACTCCCCGCGTGTGACGAGTTCCTCCCTGAGCGCGCGTGTAGGCTGGAGCTGCTTC	
dDf	GACATGAAGGACGTCGAAGAGGTCACAGCGGCGGACATGATTCCGGGGATCCGTCGACC	lmbD
dDr	GTTCACCGCTCCCGCGTGAACCGCGCGCCCACGGCCCTATGTAGGCTGGAGCTGCTTC	
dEf	CGAACAAGGGAAAGTAAGGAACGAGACATGACTCAGTGCATTCCGGGGATCCGTCGACC	lmbE
dEr	TCACGCGGGAGCGGTGAACAGGTCGTGCACCACGGCTTCTGTAGGCTGGAGCTGCTTC	
dFf	GCTCGGCACGGCGGGGGCGACCCGATGACCGCCACGGCGATTCCGGGGATCCGTCGACC	lmbF
dFr	TTTCCCTTGTTCGATTCGCTGGGTGGGCGGCCGTCACCGTGTAGGCTGGAGCTGCTTC	
dIHf	GCCGCTACCACGTGTCCGTCTACGCCCGGGCGCACGGTGATTCCGGGGATCCGTCGACC	lmbIH
dIHr	GTGAAACGTCGTGTGCAACGGGCGGTTGTGGGGTGGTCATGTAGGCTGGAGCTGCTTC	
dNf	CACCGCCGCCGACACCCTCACCTGGGAGAAACCGTGAGCATTCCGGGGATCCGTCGACC	lmbN
dNr	TGAACCCGGCTCCGCCGGTGACGCAGTACCGCCCGCTCATGTAGGCTGGAGCTGCTTC	
N-dIDf	CCGGCCGCCATCGCCGCGACGGTGGCCCGTATCACCCATTGAATTCCGGGGATCCGTCGACC	*lmbN* 3’ terminal part coding for ID (in combination with dNr)
N-dCPr	GGACAGGAGGTTCCTCACAACACTCACAGGGGTCTCCTCTGTAGGCTGGAGCTGCTTC	*lmbN* 5’ terminal part coding for CP (in combination with dNf)
dQf	GGCCGACGCCGCGGTCGACAGGCTGGCGGAGGTGTCATGATTCCGGGGATCCGTCGACC	lmbQ

dQr	GAGCGAGGAAGCTCGGAGGTGCTGAGCGGGGGCGGGCTATGTAGGCTGGAGCTGCTTC	
dTf	GGCGCCCAGGTGGCTGCCGGCGGCGTGGCCGGCTATGTGATTCCGGGGATCCGTCGACC	lmbT
dTr	GGCGTCGGCCAACTCCCGGACGTTGGCGGCGAAGTCGAATGTAGGCTGGAGCTGCTTC	
dVf	CCCGGAAGCTGGGAGAGAGCCATTTACGCCATCGAGTTGATTCCGGGGATCCGTCGACC	lmbV
dVr	CGAAGGCGAGCGCGCGCCCCCGTTCCGTCCGCGCCGTCATGTAGGCTGGAGCTGCTTC	
**B) Checking primers**		
chCf	GGTGATACCGCCATGTC	lmbC
chCr	GTCCCGTATCTGCGACT	
chDf	AACGGAGGACGACATGA	lmbD
chDr	TCACGTGACCCAAGTACC	
chEf	CCAGCGAATCGAACAAG	lmbE
chEr	GGGCTGGGAGTACCTGT	
chFf	GAACGGTACGTGCAACT	lmbF
chFr	AGCAGGCACTGAGTCAT	
chIHf	GCAACGCCTCCCACACCAACG	lmbIH
chIHr	ACACGGAGCTGGACCTGATGG	
chNf	ACCGTCACCCGCCAGGAGTT	lmbN
chNr	AGCCCGTGAGCTCTTCGGTG	
chQf	TGCCGGCAGCCACTGTTCCACC	lmbQ
chQr	CCGACGAGCGGCGGAGGATGC	
chTf	GCCGAACTCGTACTGACC	lmbT
chTr	GCGGGCGAACCTCA	

chVf	GCCCCGACGGATAGT	lmbV
chVr	AGCAGATTCCGCACATAG	
**C) Primers for heterologous expression of *S. lincolnensis* genes**		
**Primer**	**Sequence (5' to 3')**	
ExN_NHisF	CCGGAATTCGGATCCGGTGAGCACTCTGGACGAGG	
ExN_NHisR	CCGAAGCTTATGCCGGCTCCCCCGC	
ExN_CHisF	CCGGAATTCATATGAGCACTCTGGACGAGG	
ExN_CPR	CCGCTCGAGATGGGTGATACGGGCCAC	
ExN_IDF	CCGCATATGAGTGTTGTGAGGAACCTCC	
ExN_CHisR	CCGCTCGAGTGCCGGCTCCCCCGC	
slpfor	CCGCATATGATCGAGGAACTCCTGCC	
slp1rev	CCGCTCGAGTTAGGGATGCGGCACGGTCACG	
slp2rev	CCGCTCGAGGGGATGCGGCACGGTCACG	
**D) Primers used in search for *slp* gene**		
P1	CGGCATGGTCGGCTCCHTNACNCAYTG	
P3-2	TGGGTCAGCGGGTACCANRCYTTRWA	
slpup	CTTCGAGGAGGTGTCGNTNGGNTAYCC	
slpdow	CGACCGCGAACGCACCGCNGTNCARGC	
**E) Primers for construction of *S. coelicolor* M145 mutant strains**		
ExNStopF	TTCCATATGTGAACTCTGGACGAGGTCCT	
ExN_coelR	CCGCTCGAGTCAGTGGTGGTGGTGGTGGTGTGCCGGCTCCCCCGC	
