# Supplementary material for: Lincosamide Synthetase—A Unique Condensation System Combining Elements of Nonribosomal Peptide Synthetase and Mycothiol Metabolism
Source: PLoS One. 2015 Mar 5;10(3):e0118850. doi: 10.1371/journal.pone.0118850 (PMC4351081; doi:10.1371/journal.pone.0118850)
Supplement: S2 Table — (PDF) [file pone.0118850.s004.pdf]

Table S2. Details of protocol for LC-MS analysis of lincomycin, methylthiolincosamide and 4-propyl-L-proline in cultivation broth.

A. Sample preparation by solid phase extraction			
**Conditions/steps/parameters**	**Lincomycin**	**Methylthiolincosamide**	**4-propyl-L-proline**
Sorbent	HLB 3cc (60 mg)	MCX 3cc (60 mg)	No extraction
Conditioning	3 mL methanol	3 mL methanol	
Equilibration	3 mL water	3 mL 2% formic acid	
Sample volume and pH<br>agent used for pH adjustment	3 mL; pH 9.0<br>ammonium hydroxide	3 mL; pH 2.5<br>formic acid	
Washing	3 mL water	3 mL 2% formic acid	
Elution	1.5 mL methanol	1.5 mL 50% methanol with 2% ammonium hydroxide	
Reconstitution	600 µL 50% methanol	300 µL; 50% methanol	
Pre-concentration	5 ×	10 ×	1 ×
B. UHPLC conditions			
Mobile phase A (aqueous)	1 mM ammonium formate, pH 9.0	0.1% formic acid	0.1% formic acid
Mobile phase B (organic)	acetonitrile	methanol	methanol
Linear gradient (min/%B)	0/5, 1.5/5, 12.5/58, 12.5/100, 14.5/100, 14.5/5	0/5, 1.0/5, 2.5/99, 4.5/99, 4.5/5	0/5, 5.0/5, 10.0/20, 10.0/99, 11.5/99, 11.5/5
Total analysis time	16 min	6 min	13 min
C. Internal standard, calibration, LOD and LOQ			
Internal standard	N-demethyllincomycin, 100 ng mL^{-1}	No quantification	4-butyl-L-proline, 100 ng mL^{-1}
Calibration (linear range)	50 - 1000 ng mL^{-1}, 5 points		5 - 250 ng mL^{-1}, 6 points
Correlation coefficient	0.9979		0.9994
LOD*	2 ng mL^{-1}		1 ng mL^{-1}
LOQ*	10 ng mL^{-1}		5 ng mL^{-1}

*LOD – limit of detection, LOQ – limit of quantification; pre-concentration included (the LOD and LOQ concentrations refer to concentrations in the supernatant of culture broth). LOD determined as a signal three times as high as the noise; LOQ determined as the lowest point of the calibration curve, confirmed by a triplicate of QC samples where the analyte concentration was determined with accuracy and precision in the range 80–120%.
