# Supplementary material for: Lincosamide Synthetase—A Unique Condensation System Combining Elements of Nonribosomal Peptide Synthetase and Mycothiol Metabolism
Source: PLoS One. 2015 Mar 5;10(3):e0118850. doi: 10.1371/journal.pone.0118850 (PMC4351081; doi:10.1371/journal.pone.0118850)
Supplement: S3 Table — (PDF) [file pone.0118850.s005.pdf]

Table S3. Details of production of recombinant LmbN, LmbN-CP and LmbN-ID

Construct No.	Primer pair*)	Vector resistance	Protein (His-tag)	Usage	Postinduction T/ time	GroES GroEL
1	ExN_NHisF / ExN_NHisR	pETDuet-1 ampicilin	LmbN (N His6)	Ab	37°C/2 h	yes
2	ExN_CHisF / ExN_CHisR	pET42b kanamycin	LmbN (C His8)	MW	25°C/5 h	yes
3	ExN_CHisF / ExN_CPR	pET42b kanamycin	LmbN-CP (C His8)	MW PPT	27°C/4 h	no
4	ExN_IDF / ExN_CHisR	pET42b kanamycin	LmbN-ID (C His8)	MW	27°C/4 h	yes

*) The primer sequences are sumarized in Table S1

chDNA = chromosomal DNA; N His6 = N-terminal 6x His-tag; C His8 = C-terminal 8x His-tag; Ab – template for production of polyclonal antibody; Mw – molecular weight standard for immunodetection; PPT – phosphopantetheinylation assays; LmbN-CP – stand-alone CP domain; LmbN-ID – stand-alone isomerase domain; GroES and GroEL – co-expression with chaperonins GroES and GroEL; All vectors are from Novagen
